# Generation of an Iba1-EGFP Transgenic Rat for the Study of Microglia in an Outbred Rodent Strain

**DOI:** 10.1523/ENEURO.0026-21.2021

**Published:** 2021-09-07

**Authors:** Jonathan W. VanRyzin, Sheryl E. Arambula, Sydney E. Ashton, Alexa C. Blanchard, Max D. Burzinski, Katherine T. Davis, Serena Edwards, Emily L. Graham, Amanda Holley, Katherine E. Kight, Ashley E. Marquardt, Miguel Perez-Pouchoulen, Lindsay A. Pickett, Erin L. Reinl, Margaret M. McCarthy

**Affiliations:** 1 Department of Pharmacology, University of Maryland School of Medicine, Baltimore, Maryland 21201; 2 Program in Neuroscience, University of Maryland School of Medicine, Baltimore, Maryland 21201

**Keywords:** brain, EGFP, Iba1, macrophage, microglia, transgenic rat

## Abstract

Neuroscience has been transformed by the ability to genetically modify inbred mice, including the ability to express fluorescent markers specific to cell types or activation states. This approach has been put to particularly good effect in the study of the innate immune cells of the brain, microglia. These specialized macrophages are exceedingly small and complex, but also highly motile and mobile. To date, there have been no tools similar to those in mice available for studying these fundamental cells in the rat brain, and we seek to fill that gap with the generation of the genetically modified Sprague Dawley rat line: *SD-Tg(Iba1-EGFP)Mmmc*. Using CRISPR-Cas/9 technology, we knocked in *EGFP* to the promoter of the gene *Iba1*. With four male and three female founders confirmed by quantitative PCR analysis to have appropriate and specific insertion, we established a breeding colony with at least three generations of backcrosses to obtain stable and reliable Iba1-EGFP expression. The specificity of EGFP expression to microglia was established by flow cytometry for CD45^low^/CD11b^+^ cells and by immunohistochemistry. Microglial EGFP expression was detected in neonates and persisted into adulthood. Blood macrophages and monocytes were found to express low levels of EGFP, as expected. Last, we show that EGFP expression is suitable for live imaging of microglia processes in acute brain slices and via intravital two-photon microscopy.

## Significance Statement

Neuroscience research in rat models has lagged compared with the mouse because of limitations in the ability to generate genetic modifications. To fill part of that gap, we have generated a transgenic rat in which the innate immune cells of the brain, microglia, express EGFP. This modification allows for isolation of microglia from other cells by flow cytometry or FACS for detailed transcriptomic and proteomic analysis. The visualization of EGFP in acute brain slices or by *in vivo* imaging further enhances the ability to interrogate the role of these critical cells across the life span and in health and disease.

## Introduction

Microglia are the resident macrophages of the brain. Despite comprising only 10–15% of the total number of cells (Lawson et al., 1990), microglia have a drastic impact on the brain throughout the life span (Bilbo and Schwarz, 2012). During development, these specialized phagocytes act as “sculptors” of the brain by regulating cell number, coordinating synaptic connectivity, and facilitating myelination (Sierra et al., 2010; Paolicelli et al., 2011; Schafer et al., 2012; Lenz et al., 2013; Parkhurst et al., 2013; Ueno et al., 2013; Hagemeyer et al., 2017; VanRyzin et al., 2019). As the brain matures, microglia function and phenotype shift; by adulthood, microglia are tiled throughout the brain where they can effectively survey their local environment, facilitate synaptic transmission, and respond to injury (Davalos et al., 2005; Nimmerjahn et al., 2005; Wake et al., 2009; Rogers et al., 2011; Nikodemova et al., 2015).

Given the variable functional repertoire and the dynamic nature of these cells, the ability to faithfully identify and label microglia is of upmost importance to researchers. Changes in microglia function are often reflected as changes in morphology or the number at a given point in time and are often studied by examining aspects of microglia morphology in relation to other end points of interest (i.e., phagocytic cups, synaptic contacts, process motility). Identifying microglia morphology is usually accomplished by using antibody labeling for immunohistochemical analysis. Microglia express a number of proteins that can distinguish them from surrounding neural tissue, the most common being ionized calcium-binding adapter molecule 1 [Iba1; also called allograft inflammatory factor-1 (Aif1)], which provides robust histologic labeling and has been widely used to good effect for many years. However, immunohistochemical analysis is severely limited as it is a postmortem method of analysis and does not allow for direct assessments of microglia function in real time.

The tools and techniques for microglia analysis have rapidly evolved for researchers using the mouse as a model organism (Guttenplan and Liddelow, 2019). These include several knock-in models such as *CX3CR1* (the fractalkine receptor) being replaced with *EGFP* (Jung et al., 2000), *EGFP* driven by the endogenous *Iba1* promoter (Hirasawa et al., 2005), and, more recently, *EGFP* driven by the microglia-specific *Tmem119* gene (Kaiser and Feng, 2019). These tools have revealed unexpected roles for microglia in regulating synapses (Paolicelli et al., 2011; Schafer et al., 2012; Parkhurst et al., 2013), astroglial transitioning in the subventricular proliferative zone (Xavier et al., 2015), and distinguishing infiltration of peripheral macrophages from endogenous microglia following brain injury (Tanaka et al., 2003), to name very few. The development of comparable resources for rats has been minimal and thereby hampered progress in this valuable animal model.

Rats provide some distinct advantages over the mouse, not the least of which is physical size, which can be a limiting factor when working with very young animals or collecting small tissue samples for downstream processing [e.g., flow cytometry, fluorescence activated cell sorting (FACS), proteomics]. Many laude the rat for superior cognitive ability and social behavior complexity (Ellenbroek and Youn, 2016), and, although this notion has been challenged (Jaramillo and Zador, 2014), there is still much to understand given the importance of microglia in regulating these behavioral domains (Frost and Schafer, 2016). Rats also exhibit some behaviors not readily apparent in the mouse, for example, juvenile rough-and-tumble play (VanRyzin et al., 2019). Many sex differences in the rat brain are determined by the developmental actions of microglia (Lenz and Nelson, 2018), but a similar relationship has not yet been established in the mouse.

To address this shortcoming and provide a resource for investigators wishing to study microglia while capitalizing on the advantages of using a rat model, we contracted the design and development of a novel *Iba1-EGFP* transgenic rat. We show that EGFP expression is robust, highly specific to microglia in the brain, and is suitable for cell-sorting and live-imaging studies.

## Materials and Methods

### Generation and validation of SD-Tg (Iba1-EGFP)Mmmc transgenic rats

Applied StemCell was contracted to generate the Iba1-EGFP knock-in rat model using CRISPR/Cas9 technology in the Sprague Dawley rat strain. The donor construct inserted consisted of the *EGFP* coding sequence (minus the first ATG), followed by the 22 aa sequence of the porcine teschovirus-1 2A (P2A) self-cleaving peptide and then the first exon of the rat *Iba1* gene immediately downstream of the translational start site. Guide RNA candidates targeting the *Iba1* gene just downstream of the translational start site were prepared via *in vitro* transcription from a T7 promoter and individually tested for efficiency in Sprague Dawley embryos. The guide RNA that was used demonstrated 100% efficiency and had the following sequence: 5′-TACCCTGCAAATCCTTGCTCTGG-3′. Microinjected embryos were implanted into Sprague Dawley surrogate dams and the resulting pups were screened for site-specific insertion of *EGFP* sequence via PCR. Of 34 pups screened, 7 were positive for *EGFP* sequence with the correct 5′ and 3′ insertion sites at the endogenous *Iba1* sequence. PCR products from these animals were sequenced to further confirm site-specific insertion of *EGFP* downstream of the *Iba1* translational start site, the formation of correct junctions, and the fidelity of the sequence contained within the insertion. Two heterozygous male and three heterozygous female founder animals were shipped to the University of Maryland School of Medicine and were maintained on a 12 h reverse light/dark cycle with *ad libitum* access food and water. Animals were mated in our facility, and pregnant females allowed to deliver naturally with the day of birth designated as postnatal day 0 (P0). Male and female F2 and F3 offspring of founder animals were used in these studies. All animal procedures were performed in accordance with the regulations of the Animal Care and Use Committee at the University of Maryland School of Medicine. This transgenic line has been registered with the Medical College of Wisconsin Rat Resource Center as SD-Tg(Iba1-EGFP)Mmmc.

### Genotyping

Genotyping of *Iba1-EGFP* offspring was performed using MyTaq Extract-PCR reagents (Meridian Bioscience). Tail snips taken from pups during the first 5 postnatal days or ear clips from adult animals were used to provide DNA for genotyping. Tissue homogenates were diluted 1:100 in double-distilled H_2_O and were used in a PCR containing 200 nm each primer, and thermocycled through 95°C for 60 min, 55°C for 30 s, and 72°C for 90 s for 34 cycles. PCR primers for genotyping were designed to target the 5′ upstream regulatory sequence of the endogenous *Iba1* gene and the *Iba1* coding sequence, and span the insertion site of the *EGFP* sequence (Integrated DNA Technologies; Table 1). Thus, both the wild-type *Iba1* allele and the *EGFP* insertion were detected as bands of 334 and 1114 bp, respectively. Genotyping was confirmed and is commercially available from TransnetYX.

**Table 1:** List of RNA sequences and primers

Target gene	Forward sequence (5′ > 3′)	Reverse sequence (5′ > 3′)	Amplicon size (bp)	Accession number	Usage
*Iba1*	AGCCAGAGCAAGGATTTGCAG	N/A	N/A	NM_017196.3	Guide RNA
*Iba1*	N/A	TACCCTGCAAATCCTTGCTCTGG	N/A	NM_017196.3	Guide RNA
*EGFP-Iba1*	GACAATATGCGCCTGGACAA	GTGCTGTACGGTCCACCTTC	334 (WT) or 1114 (insert)	N/A	Genotyping
*CX3CR1*	TGCTCAGGACCTCACCATGC	AAGATGGTCCCAAAGGCCAC	117	NM_133534.1	qPCR
*EGFP*	CCCGACAACCACTACCTGAG	GTCCATGCCGAGAGTGATCC	117	N/A	qPCR
*Gapdh*	TCCAGTATGACTCTACCCACG	CACGACATACTCAGCACCAG	149	NM_017008.4	qPCR
*Gfap*	GCGAAGAAAACCGCATCACC	TTTGGTGTCCAGGCTGGTTT	77	NM_017009.2	qPCR
*Iba1*	TGGAGTTTGATCTGAATGGCAATG	AGCCACTGGACACCTCTCTA	126	NM_017196.3	qPCR
*Olig2*	GAACCCCGAAAGGTGTGGAT	TTCCGAATGTGAATTCGATTTGAGG	106	NM_001100557.1	qPCR
*Nes*	CAGAGAAGCGCTGGAACAGA	CCACAGCCAGCTGGAACTTA	80	NM_012987.2	qPCR
*Neun*	CGTTCCCACCACTCTCTTGT	TAGCCTCCATAAATCTCAGCACC	82	NM_001134498.2	qPCR

N/A, Not applicable.

### Western blot

Total protein was isolated from cortical tissue of EGFP^+/+^, EGFP^–/+^, and EGFP^−/−^ (wild-type) rats at P18 by homogenizing in 10 mm Tris-HCl, pH 7.6/150 mm NaCl/1% Nonidet P-40/1% sodium deoxycholate, with protease and phosphatase inhibitors (Sigma-Aldrich). For each sample, 30 μg of cortical protein homogenate was resolved on a 12% Bis-Tris polyacrylamide gel (NuPage, Thermo Fisher Scientific) under reducing and denaturing conditions, and transferred to PVDF membrane. Membranes were blocked with Odyssey Blocking Buffer (LI-COR) diluted 1:1 with Tris-buffered saline (TBS), pH 7.4, and incubated overnight at 4°C with primary antibodies diluted in a 1:1 solution of Blocking Buffer/TBS-0.01% Tween. The primary antibodies used were chicken-anti-GFP (1:4000; Thermo Fisher Scientific) and mouse anti-Gapdh (1:5000; Abcam). After washing in TBS-0.1% Tween, membranes were incubated in 1:1 Blocking Buffer–TBS-Tween/0.02% SDS containing secondary antibodies (both diluted 1:10,000; IRDye 800 donkey-anti-chicken and IRDye 680 goat-anti-mouse IgG, LI-COR). Membranes were imaged on an Odyssey CLx Infrared Imaging System (LI-COR).

### Flow cytometry

Animals were deeply anesthetized with Fatal Plus (Vortech Pharmaceuticals) and were transcardially perfused with ice-cold PBS, 0.1 m, pH 7.4, until the perfusate was clear. Intracardiac blood was collected, washed with PBS, resuspended in an ammonium chloride red blood cell lysis buffer for 10 min, and centrifuged to obtain a clear cell pellet before antibody labeling. Neonatal (P7) brains, spinal cords, and dorsal root ganglia were removed and dissociated with the Neural Tissue Dissociation Kit P (Miltenyi Biotec). Adult (P60+) brains were dissociated with 1 mg/ml collagenase-D (Sigma-Aldrich) and 0.25 mg/ml DNase I (Sigma-Aldrich) in RPMI solution (Thermo Fisher Scientific) for 20 min. Cells were washed in RPMI solution, resuspended in 37% Percoll (Sigma-Aldrich), and centrifuged at 1200 × *g* for 15 min to separate the myelin debris layer. Both neonatal and adult brain homogenates were washed with ice-cold FACS buffer (2% bovine serum albumin, 2 mm EDTA in PBS) before antibody labeling.

Blood and brain samples were blocked with anti-CD32 (1:100; clone D34-485, BD Biosciences) and stained with Fixable Viability Dye eFluor 780 (Thermo Fisher Scientific). To identify microglia, cells were labeled with anti-CD11b-PE (1:200; clone WT.5, BD Biosciences) and anti-CD45-AF700 (1:200; clone OX-1, BD Biosciences) antibodies in FACS buffer. Cells were washed and analyzed on an LSRII flow cytometer (BD Biosciences) with FACSDiva software.

To characterize peripheral immune cells, cells were labeled with anti-RT1B BV421 (1:200; clone OX-6, BD Biosciences), anti-CD3 PE (1:200; clone 1F4, BD Biosciences), anti-CD45R Pe-Cy7 (1:200; clone HIS24, Thermo Fisher Scientific), anti-CD11b APC (1:200; clone WT.5, BD Biosciences), anti-CD45 AL700 (1:200; clone OX-1, BD Biosciences), and anti-rat granulocyte biotin (1:200; clone His48, Thermo Fisher Scientific) followed by streptavidin PerCp-Cy5.5 (1:500; Thermo Fisher Scientific) in FACS buffer. Cells were washed and analyzed on a Cytek Aurora flow cytometer (CYTEK). Data were analyzed using FCS Express 6 (De Novo Software) and FlowJo X (FlowJo) software.

### Cell sorting

Samples were prepared as described for flow cytometry, without the addition of antibody or dye labeling and sorted using an Aria II Cell Sorter (BD Biosciences) with a 100 μm pore size. Samples were gated for EGFP expression and run until 50,000 EGFP^+^ cells were collected. Cells were then resuspended in Qiazol (Qiagen) and stored at −80°C until processed.

### RNA isolation, cDNA synthesis, and quantitative PCR

Total RNA was extracted using the protocol for fatty tissues from the RNeasy Handbook for Mini Kit (Qiagen). Single-stranded cDNA synthesis was performed with 200 ng of RNA input using the high-capacity cDNA Reverse Transcription Kit (Thermo Fisher Scientific), and samples were stored at −20°C until use. Quantitative PCR (qPCR) was performed using an Applied Biosystems ViiA7 PCR System (Thermo Fisher Scientific) with the following cycling parameters: 50°C for 2 min, 95°C for 10 min, followed by 40 cycles of 95°C for 15 s and 60°C for 1 min. Primers (Integrated DNA Technologies; Table 1) were designed using Primer3 software, and primer efficiency was determined through the use of serial dilutions. Samples were run in triplicate, cycle threshold (Ct) values for the gene of interest were normalized to the Ct for *Gapdh* (Δ-Ct), and relative data were determined by the ΔΔ-Ct method (Schmittgen and Livak, 2008).

### Immunohistochemistry

Animals were deeply anesthetized with Fatal Plus (Vortech Pharmaceuticals) and transcardially perfused with PBS, 0.1 m, pH 7.4, followed by 4% paraformaldehyde (PFA; 4% in PBS), pH 7.2. Brains were removed and postfixed for 24 h in 4% PFA at 4°C, then kept in 30% sucrose at 4°C until fully submerged. Coronal sections (45 μm thick) were cut on a cryostat (model CM2050S, Leica) and directly mounted onto slides. Slide-mounted sections were washed in PBS, blocked with 5% normal goat serum (NGS) in PBS + 0.4% Triton X-100 (PBS-T) for 1 h, and incubated with anti-GFP (1:1000; catalog #ab13970, Abcam) and anti-Iba1 (1:1000; catalog #019–19 741, Wako) in 2% NGS in PBS-T overnight. The next day, slides were incubated with Alexa Fluor 488 (1:500; Thermo Fisher Scientific) and Alexa Fluor 594 (1:500; Thermo Fisher Scientific) in PBS-T for 2 h, washed and stained with Hoechst 33342 (1:3000; catalog #H3570, Thermo Fisher Scientific) for 10 min, and coverslipped with ProLong Diamond Antifade (Thermo Fisher Scientific).

### Microscopy and colocalization analysis

Wide-field fluorescence images were captured on a Keyence BZ-X700 microscope using a 10× objective [0.45 numerical aperture (NA)] and 20× objective (0.75 NA) and BZ-X Viewer software. For colocalization analysis, single field-of-view images were taken at 20× magnification using 0.4 μm *z*-steps through the entire tissue thickness. Subsequent maximum intensity projections were used to quantify microglia as Iba1^+^ and EGFP^+^ using the cell counter plugin in Fiji (Schindelin et al., 2012).

### Slice preparation and live imaging

The brain was rapidly dissected out from a P7 rat pup following decapitation and immediately placed in ice-cold artificial CSF (aCSF) containing (in mm) 125 NaCl, 2.5 KCl, 1 MgCl_2_, 1.25 NaH_2_PO_4_, 2 CaCl_2_, 25 NaHCO_3_, 25 glucose, and 75 sucrose, pH 7.4 (Dailey et al., 2013; Huang et al., 2018). Coronal sections (∼0.5 mm) were cut using a Zivic Brain Slicer Matrix on ice. A section was transferred to a MaTek glass bottom microwell dish (35 mm dish, no. 1.5 coverslip) containing room temperature aCSF (in mm: 125 NaCl, 2.5 KCl, 1 MgCl_2_, 1.25 NaH_2_PO_4_, 2 CaCl_2_, 25 NaHCO_3_, and 10 glucose, pH 7.4). Confocal fluorescent images using a 1 μm *z*-step across 10 μm of tissue for a single field of view were acquired once per minute for a total of 20 min with a Nikon A1 microscope equipped with a 488 laser using an Apo 60× Oil objective (1.4 NA). ATP (Sigma-Aldrich) was bath applied (1 mm in aCSF) after 4 min of baseline imaging. The motion of 10 microglial processes from five microglia was analyzed using the MTrackJ plugin (https://imagej.net/MTrackJ) for ImageJ (Meijering et al., 2012).

### Intravital two-photon microscopy

Neonatal (P14) *Iba1-EGFP^+/+^* rats and adult *B6.129P-CX3CR1^tm1Litt^/J* (*CX3CR1^gfp/+^* mice; gift from Bogdan Stoica, Department of Anesthesiology, University of Maryland School of Medicine, Baltimore, MD) were anesthetized with ketamine (85 mg/kg) and xylazine (13 mg/kg), and maintained on a heating pad. A thin skull preparation was performed as previously described (Roth et al., 2014). A metal bracket was secured over the parietal lobe, and the skull bone was thinned to ∼30–60 μm. Intravital two-photon microscopy was performed using a two-photon microscope (SP5 II, Leica) equipped with a 20× water-dipping objective (1.0 NA), a PMT-ready Objective Inverter (LSM TECH), and a Coherent Chameleon Laser tuned to 880 nm for GFP (provided by Alan Faden, the University of Maryland Center for Shock Trauma and Anesthesiology Research, Baltimore, MD). 3D time-lapse movies were captured in *z*-stacks of 10–15 planes (step size, 3 μm; 3× zoom) at ∼30 s intervals. Image analysis was performed using Imaris (Oxford Instruments) and ImageJ.

### Quantification and statistical analysis

Statistical analysis was performed using R (version 3.4.4; R Core Team, 2018) or GraphPad Prism 8. See Table 2 for details regarding specific data or comparisons (e.g., descriptive statistics, test used, *n*, which are referenced in text using superscript letters). Analyses were considered significant at *p *≤* *0.05.

**Table 2:** Summary of descriptive statistics and statistical analyses

Line	Data structure	Type of test	Description of data or analysis	Test value	*p* Value	Mean ± SD
a	Normal distribution	One-way ANOVA	Iba1 protein, WT (*n* = 5) vs Het (*n* = 6) vs Homo (*n* = 7)	*F*_(2,15)_ = 0.7877	*p* = 0.4728	WT: 547,400 ± 202,011 Het: 471,500 ± 268,511
						Homo: 402,143 ± 104,266
b	Normal distribution	Welch’s *t* test	qPCR quantification (ΔCt); Iba1 expression at P7 (*n* = 15), and P60 (*n* = 6)	*t*_(6.9003)_ = −2.0425	*p* = 0.081	P7: 7.84 ± 0.99 P60: 9.16 ± 1.46
c	Normal distribution	Welch’s *t* test	qPCR quantification (ΔCt); EGFP expression at P7 (*n* = 15), and P60 (*n* = 6)	*t*_(7.9695)_ = −1.5523	*p* = 0.1593	P7: 10.01 ± 1.81 P60: 11.56 ± 2.16
d	Normal distribution	Welch’s *t* test	Median fluorescence intensity; Het (*n* = 4) vs Homo (*n* = 6)	*t*_(7.5763)_ = −1.8756	*p *=* *0.09963	Het: 623.25 ± 119.96 Homo: 843.67 ± 247.54
	Normal distribution	One-sample *t* test	qPCR quantification (ΔΔCt); EGFP^+/+^ sorted cells (*n* = 3) vs normalized value = 0			
e			*Iba1*	*t*_(2)_ = −11.036	*p *=* *0.00811	19.84 ± 8.67
f			*CX3CR1*	*t*_(2)_ = −63.772	*p *=* *0.0002458	37.35 ± 3.57
g			*Nes*	*t*_(2)_ = 6.8055	*p *=* *0.02092	0.40 ± 0.09
h			*Gfap*	*t*_(2)_ = 55.708	*p *=* *0.0003221	0.08 ± 0.03
i			*Olig2*	*t*_(2)_ = 11.419	*p *=* *0.007582	0.04 ± 0.004
j			*NeuN*	*t*_(2)_ = 3.0812	*p *=* *0.09116	0.28 ± 0.17
k	Normal distribution	Unpaired *t* test	Median fluorescence intensity; blood (*n* = 9) vs brain (*n* = 9), P7	*t*_(17)_ = 2.2865	*p* ≤ 0.0001	Blood: 123.56 ± 16.49 Brain: 755.50 ± 227.59
l	Normal distribution	Unpaired *t* test	Median fluorescence intensity; blood (*n* = 6) vs brain (*n* = 6), P60	*t*_(10)_ = 5.5178	*p *=* *0.0003	Blood: 758.17 ± 161.10 Brain: 9690.2 ± 3961.8
m	Normal distribution	Unpaired *t* test	CD45 high EGFP^+^ in brain and blood	*t*_(4)_ = 5.050	*p *=* *0.0072	Blood: 15.87 ± 4.704 Brain: 52.23 ± 11.55
	Normal distribution	Unpaired *t* test	EGFP^+^ population in brain (*n* = 3) and blood (*n* = 3)			
n			B cells	*t*_(4)_ = 2.825	*p *=* *0.0476	Blood: 11.81 ± 7.18 Brain: 0.1 ± 0.17
o			T cells	*t*_(4)_ = 4.085	*p *=* *0.0150	Blood: 0.13 ± 0.06 Brain: 0.95 ± 0.34
p			Myeloid/macrophage	*t*_(4)_ = 32.20	*p* = <0.0001	Blood: 2.92 ± 0.6 Brain: 61.67 ± 3.1
q			Monocyte	*t*_(4)_ = 3.479	*p *=* *0.0254	Blood: 51.93 ± 15.12 Brain: 18.97 ± 6.39
r			Other	*t*_(4)_ = 1.979	*p *=* *0.1189	Blood: 28.1 ± 10.91 Brain: 15.03 ± 3.42
s	Normal distribution	Unpaired *t* test	Myeloid cell/macrophage EGFP^+^ in brain (*n* = 3) and blood (*n* = 3)	*t*_(4)_ = 3.999	*p *=* *0.0161	Blood: 62.17 ± 9.767; Brain: 85.37 ± 2.359
t	Normal distribution	Unpaired *t* test	Monocyte EGFP^+^ in brain (*n* = 3) and blood (*n* = 3)	*t*_(4)_ = 2.568	*p *=* *0.0621	Blood: 39.97 ± 13.79 Brain: 69.43 ± 14.31
			Iba1^+^/GFP^+^ colocalization; Het (*n* = 4) vs Homo (*n* = 6)			
u			Prefrontal cortex			Het: 93.97 ± 12.06% Homo: 100 ± 0%
v			Nucleus accumbens			Het: 91.59 ± 3.69% Homo: 98.96 ± 2.55%
w			Hippocampus			Het: 92.59 ± 9.19% Homo: 95.34 ± 8.42%
x			Amygdala			Het: 91.39 ± 5.05% Homo: 100 ± 0%

Het, Heterozygous; Homo, homozygous.

## Results

### Generation of SD-Tg (Iba1-EGFP)Mmmc transgenic rats

**Figure 1.**
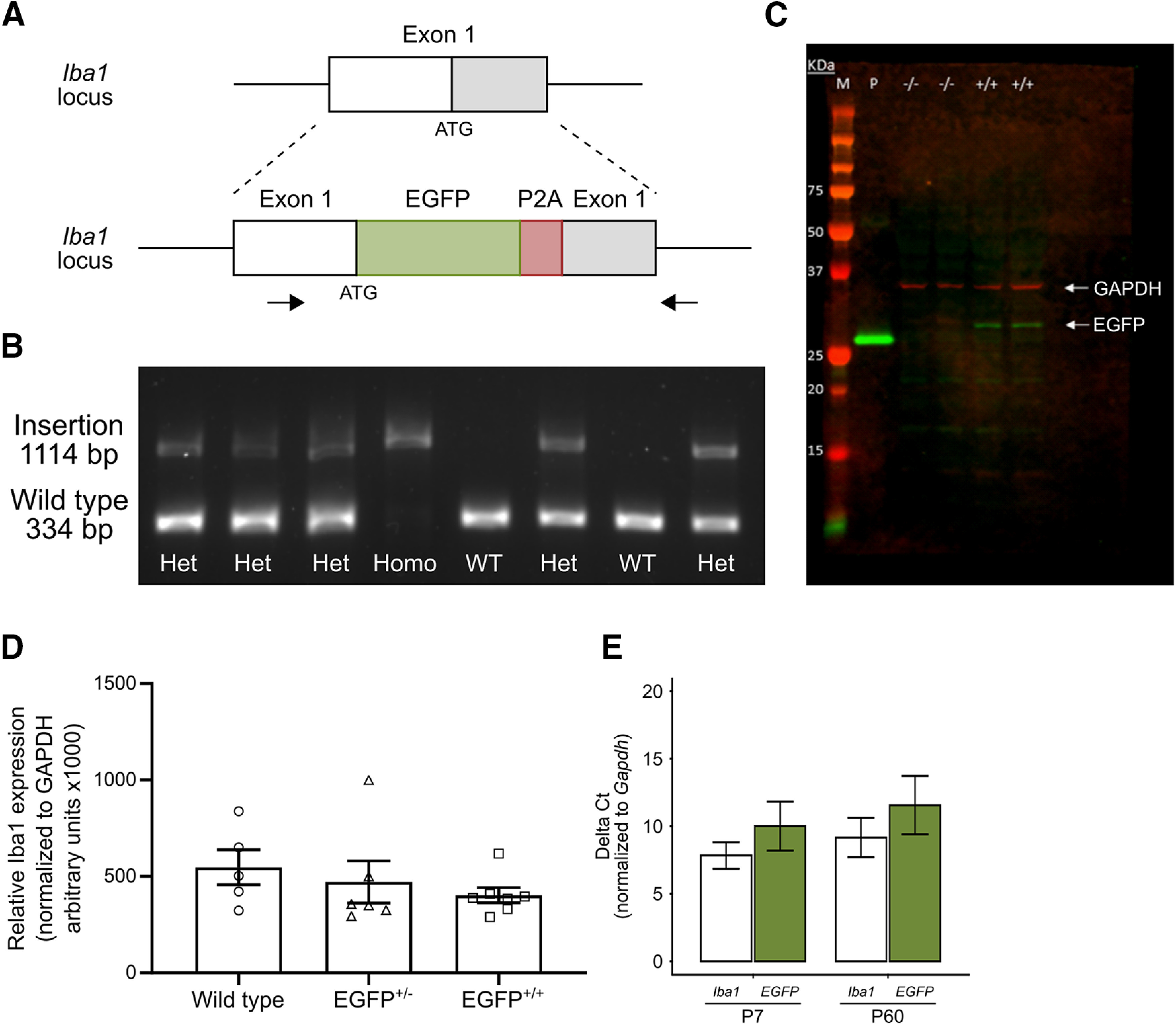
Generation of Iba1-EGFP knock-in rat. ***A***, Schematic of exon 1 of the Iba1 gene (top) and the EGFP-P2A insertion (bottom). The black bar indicates the transcription start site (ATG), and the gray region represents the protein-coding region of the exon. The EGFP (green) and P2A (red) sequences were inserted at the transcription start site of exon 1. ***B***, Representative image of a genotyping gel showing the presence of two distinct bands. The lower band (334 bp) is the amplification of the wild-type allele, while the upper band (1114 bp) is the amplification of the EGFP-P2A-containing allele. ***C***, Representative Western blot image for GAPDH protein (top band; red) and EGFP protein (bottom band; green). Lane M, Protein marker (various sizes labeled on the left); lane P, 30 ng of purified recombinant EGFP protein; lane −/−, 30 μg of total protein from cortical homogenates of EGFP^−/−^ rats; lane +/+, 30 μg of total protein from cortical homogenates of EGFP^+/+^ rats. ***D***, Quantification of Iba1 protein expression in wild-type, EGFP^+/−^, and EGFP^+/+^ littermates. ***E***, qPCR quantification of *Iba1* and *EGFP* transcripts in EGFP^+/+^ rats at various time points during development. Open symbols in ***D*** represent individual animal datapoints.

We sought to generate a model in which EGFP was expressed under control of the endogenous *Iba1* promoter without altering the endogenous Iba1 protein. To this end, the *EGFP* coding sequence and *P2A* linker sequence were inserted immediately following the translation start codon in the first exon of rat *Iba1* and upstream of the remainder of the endogenous *Iba1* gene (Fig. 1*A*). This results in the expression of both EGFP and Iba1 in a single mRNA transcript under control of the endogenous regulatory sequence of the *Iba1* gene. Ribosomal skipping at the *P2A* sequence results in a modified EGFP that contains 17 aa of the P2A peptide at the C terminus and Iba1 as the translation products. For genotyping, we designed forward and reverse primers for PCR that spanned exon 1. The resulting PCR products confirmed the presence of the *EGFP/P2A* insertion in homozygous (EGFP^+/+^) and heterozygous (EGFP^+/−^) offspring, while the insert was absent in wild-type (EGFP^−/−^) littermates (Fig. 1*B*). To verify whether the *EGFP/P2A* insertion altered expression of the *Iba1* gene, we first used Western blot to determine the presence of unspliced EGFP-P2A-Iba1 fusion proteins in cortical tissue homogenates. As expected anti-GFP antibody detected a band of ∼35 kDa, which corresponds to the EGFP protein with the 17 aa P2A peptide on the C terminus that was present in EGFP^+/+^ rats, but not in EGFP^−/−^ rats. Higher-molecular-weight bands corresponding to EGFP-P2A-Iba1 fusion protein, which is predicted at ∼52 kDa, were not detected. We then quantified Iba1 protein and found no difference in Iba1 levels across genotypes, demonstrating that the expression of Iba1 protein in transgenic animals is comparable to endogenous Iba1 expression in WT rats (Fig. 1*D*^a^). Finally, we quantified *Iba1* and *EGFP* mRNA by qPCR at both P7 and P60 to determine whether the gene expression varied with age. The mRNA levels of both Iba1 and eGFP were comparable at both ages (*p *=* *0.08^b^ for Iba1; *p *=* *0.16^c^ for EGFP; Fig. 1*E*), indicating that the transgene is robustly expressed into early adulthood.

### Flow cytometry validation of EGFP^+^ cells

**Figure 2.**
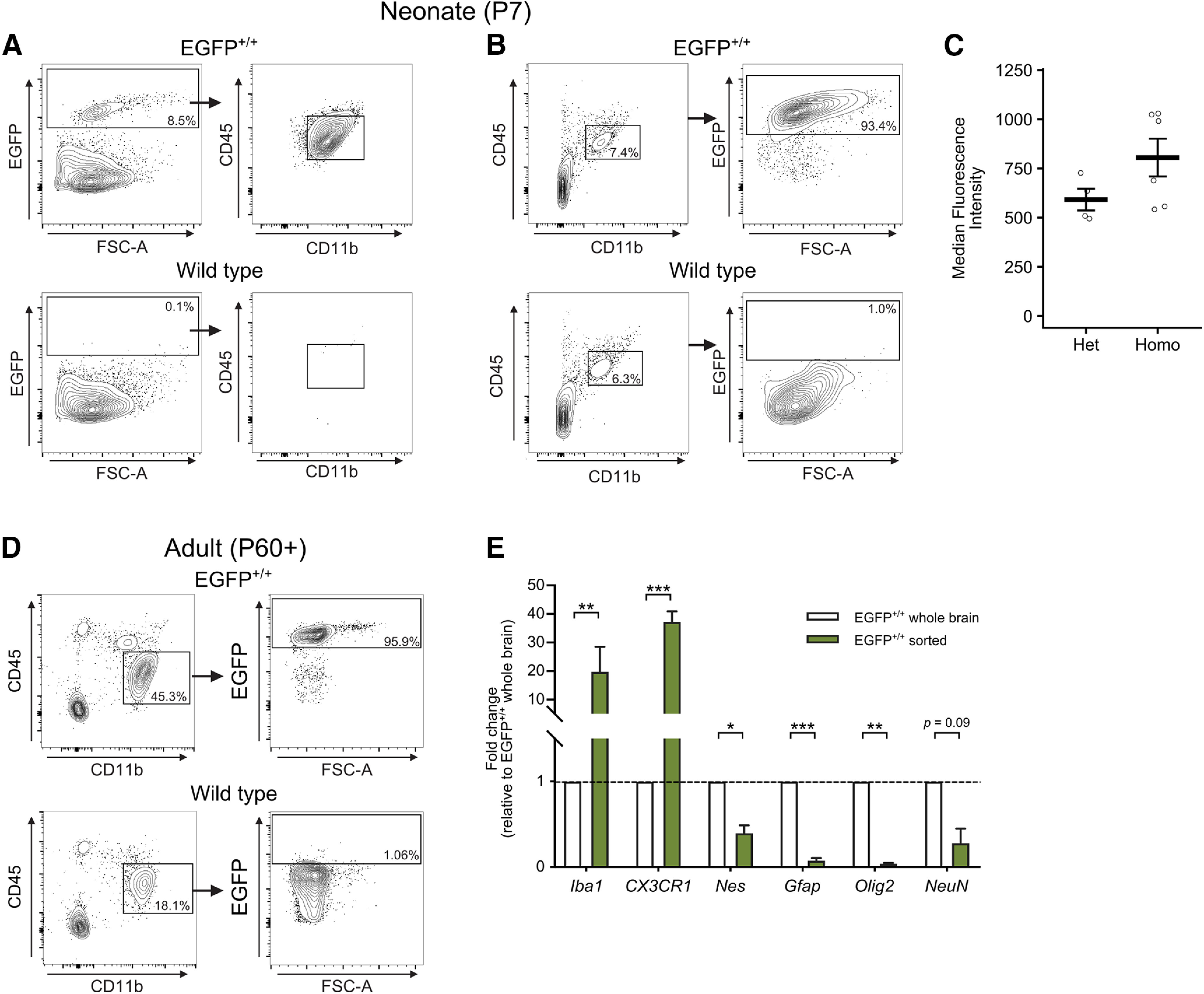
Flow cytometry validation of microglial Iba1-EGFP expression in neonates and adults. ***A***, Brain cells isolated from either EGFP^+/+^ (top) or wild-type littermates (bottom) at P7 were gated as EGFP^+^ and analyzed for their expression as CD11b^+^/CD45^int^ (right). Gated on forward scatter (FSC)/side scatter (SSC), singlets, live, CD45^int^, CD11b^+^. ***B***, The same samples from ***A*** were reanalyzed, gated as CD11b^+^/CD45^int^ (left) and analyzed for their expression as EGFP^+^ (right). In ***B*** and ***C***, the black boxes indicate gates, and the percentage of cells within each gate are shown. ***C***, Quantification of the median fluorescence intensity of EGFP^+^ cells isolated from heterozygous or homozygous littermates at P7. ***D***, Brain cells isolated from either EGFP^+/+^ (top) or wild-type littermates (bottom) at P60 or later were gated as CD11b^+^/CD45^int^ (left) and analyzed for their expression as EGFP^+^ (right). ***E***, qPCR quantification of microglial genes (*Iba1*, *CX3CR1*) and nonmicroglial genes (*Nes*, *Gfap*, *Olig2*, *NeuN*) from EGFP^+^ sorted cells or whole-brain homogenates from EGFP^+/+^ animals at P7. ***E***, Brain cells from adult EGFP^+/+^ and WT animals gated on CD11b^+^/CD45^int^ were analyzed for their expression as EGFP^+^. Histogram reflects a shift in EGFP fluorescence intensity in adult microglia-like cells in the *Iba1-EGFP* rat (green) compared with WT control (gray). Contour lines in ***A***, ***B***, and ***D*** represent 95% of the data at 5% intervals. Black boxes in ***A***, ***B***, and ***D*** indicate gates, and the percentage of cells within each gate are shown. Bars in ***C*** represent the mean ± SEM. Bars in ***E*** represent the mean ± SD. Open circles in ***C*** represent individual animal datapoints. *p < 0.05; **p < 0.01; ***p < 0.001.

To determine whether EGFP^+^ cells expressed markers commonly associated with microglia, we used flow cytometry to analyze the expression of CD45 and CD11b in cells acutely isolated from the brains of P7 EGFP^+/+^ or EGFP^−/−^ (wild-type) littermates (Fig. 2*A*,*B*). In whole-brain homogenates, ∼9.8% of cells were EGFP^+^ from EGFP^+/+^ animals compared with 0.1% of cells from wild-type animals (Table 3). Furthermore, 95.3% of the EGFP^+^ cells were identified as CD11b^+^/CD45^int^, consistent with microglia-like patterns of expression (Fig. 2*A*, Table 3).

**Table 3:** Iba1-EGFP Flow cytometry results

	Wild type	EGFP^+/+^	Figure reference
PN7	(*n* = 4)	(*n* = 6)	
EGFP^+^ cells (mean counts ± SD, % live singlets)	38 ± 1, 0.1 ± 0.0%	6039 ± 440, 9.8 ± 0.7%	Figure 2*A*
Subgate: proportion of EGFP^+^ cells that are CD11b^+^/CD45^int^ (counts ± SD, % EGFP^+^ ± % SD)	N/A	5754 ± 402, 95.3 ± 6.7%	
CD11b^+^/CD45*^int^* cells (counts/live singlets, % live singlets)	5111 ± 174, 8.3 ± 0.3%	6054 ± 324, 9.8 ± 0.5%	Figure 2*B*
Subgate: proportion of CD11b^+^/CD45^int^ cells that are EGFP^+^ (counts ± SD, % CD11b^+^/ CD45^int^ ± % SD)	33 ± 2, 0.7 ± 0.0%	5745 ± 402, 94.9 ± 6.6%	
Adult	(*n* = 2)	(*n* = 3)	
EGFP^+^ cells (mean counts ± SD, % live singlets)	594 ± 291, 0.55 ± 0.35%	25,270 ± 11,127, 37.6 ± 23.6%	N/A
Subgate: proportion of EGFP^+^ cells that are CD11b^+^/CD45^int^ (counts ± SD, % EGFP^+^ ± % SD)	N/A	22,766 ± 10,203, 89.8 ± 1.46%	
CD11b^+^/CD45^int^ cells (counts/live singlets, % live singlets)	21,995 ± 5699, 19.3 ± 1.73%	23,892 ± 10,768, 36 ± 22%	Figure 2*D*
Subgate: proportion of CD11b^+^/CD45^int^ cells that are EGFP^+^ (counts ± SD, % CD11b^+^/CD45^int^ ± % SD)	210 ± 28, 0.97 ± 0.1%	22,624 ± 10,085, 94.8 ± 1.4%	

We then analyzed the data in reverse, first gating for CD11b^+^/CD45^int^ cells and then by EGFP revealing that ∼10% of all cells were CD11b^+^/CD45^int^ in EGFP^+/+^ animals similar to age-matched wild types (8.3% of cells). Further analysis of the CD11b^+^/CD45^int^ fraction found that 94.9% were EGFP^+^ in EGFP^+/+^ compared with 0.7% in wild-type animals (Fig. 2*B*, Table 3). As the *EGFP* gene dosage may affect the relative fluorescence intensity between EGFP^+/+^ and EGFP^+/−^ animals, we compared the median fluorescence intensity (MFI) of heterozygous and homozygous littermates at P7. Overall, the MFI values were not significantly different between the two genotypes (*p *=* *0.1^d^; Fig. 2*C*). In adult rats (>P60), 94.8% of CD11b^+^/CD45^int^ cells were EGFP^+^ in EGFP^+/+^ animals compared with 0.97% in age-matched wild types, consistent with our findings in neonates and demonstrating that transgene expression is robust and maintained into early adulthood (Fig. 2*D*, Table 3). Together, these data demonstrate that nearly all of the EGFP^+^ cells in the brain express microglial markers, and of all the possible microglial cells, the vast majority are EGFP^+^ in both neonates and adults.

To further verify the identity of EGFP^+^ cells as microglia, we isolated EGFP^+^ cells from EGFP^+/+^ animals by FACS and used PCR to compare the relative expression of microglia-enriched genes (*Iba1*, *CX3CR1*) and nonmicroglial genes (*Nes*, *Gfap*, *Olig2*, *NeuN*) between EGFP^+^ sorted cells and whole-brain homogenates from EGFP^+/+^ littermates. Both *Iba1* (*p *=* *0.008^e^) and *CX3CR1* (*p *<* *0.001^f^) were greatly enriched in the EGFP^+^ sorted cell population, while markers for progenitors (Nes; *p *=* *0.02^g^), astrocytes (*Gfap*; *p *<* *0.001^h^), oligodendrocytes (*Olig2*; *p* = 0.007^i^), and neurons (*NeuN*; *p *=* *0.09^j^) had far lower expression in EGFP^+^ sorted cells compared with unsorted whole-brain homogenates (Fig. 2*E*). These data confirm EGFP expression is highly specific to microglia in the brain.

**Figure 3.**
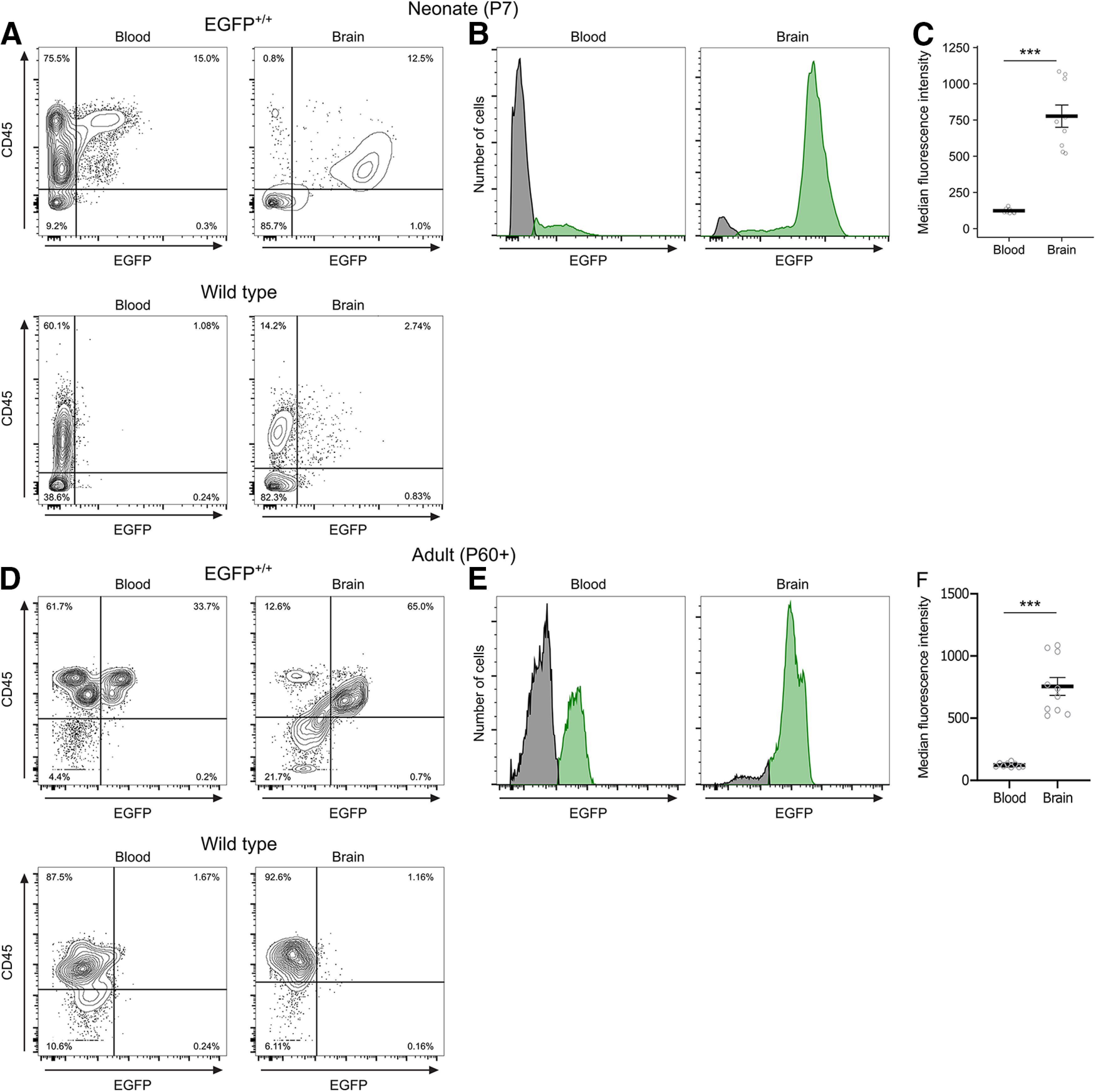
Comparison of EGFP signal in blood and brain. ***A***, Representative contour plots showing the gating strategy for median fluorescence intensity analysis in ***B*** and ***C***. Cells were gated as EGFP^+^ and CD45^+^ in samples from blood (left) or brain (right) at P7 from either EGFP^+/+^ (top) or wild-type littermates (bottom). ***B***, Representative histograms showing the distribution of EGFP fluorescence intensity of all CD45^+^ cells at P7 in the EGFP^+/+^ rat (green) compared with wild-type controls (gray) in the blood and brain. ***C***, Quantification of median fluorescence intensity at P7. ***D***, Representative contour plots showing the gating strategy for median fluorescence intensity analysis in ***E*** and ***F***. Cells were gated as EGFP^+^ and CD45^+^ in samples from blood (left) or brain (right) in adults >P60 from either EGFP^+/+^ (top) or wild-type littermates (bottom). ***E***, Representative histogram showing the distribution of EGFP^+^ fluorescence intensity of all CD45^+^ cells in the adult EGFP^+/+^ rat (green) compared with wild-type controls (gray) in the blood and brain. ***F***, Quantification of median fluorescence intensity in adults at P60 or later. Contour lines in ***A*** represent 95% of the data at 5% intervals. Bars in ***C*** and ***F*** represent the mean ± SEM. Open circles represent individual animal datapoints.

As both microglia and peripheral myeloid cells express *Iba1*, we compared EGFP expression in blood and brain samples from EGFP^+/+^ and wild-type neonates and adults using flow cytometry. We gated based on CD45 and EGFP expression and detected a distinct EGFP^+^ population in the blood of EGFP^+/+^ animals in both ages (Fig. 3*A*,*D*). EGFP^+^ cells in the blood were mostly CD45^high^, whereas in the brain, EGFP^+^ cells were CD45^int^; and directly comparing the fluorescence intensity between the two populations found that brain EGFP^+^ cells had a significantly greater MFI than EGFP^+^ cells in the blood at both ages [neonates, *p *<* *0.001^k^ (Fig. 3*B*,*C*); adults, *p *<* *0.001^l^ (Fig. 3*E*,*F*)].

**Figure 4.**
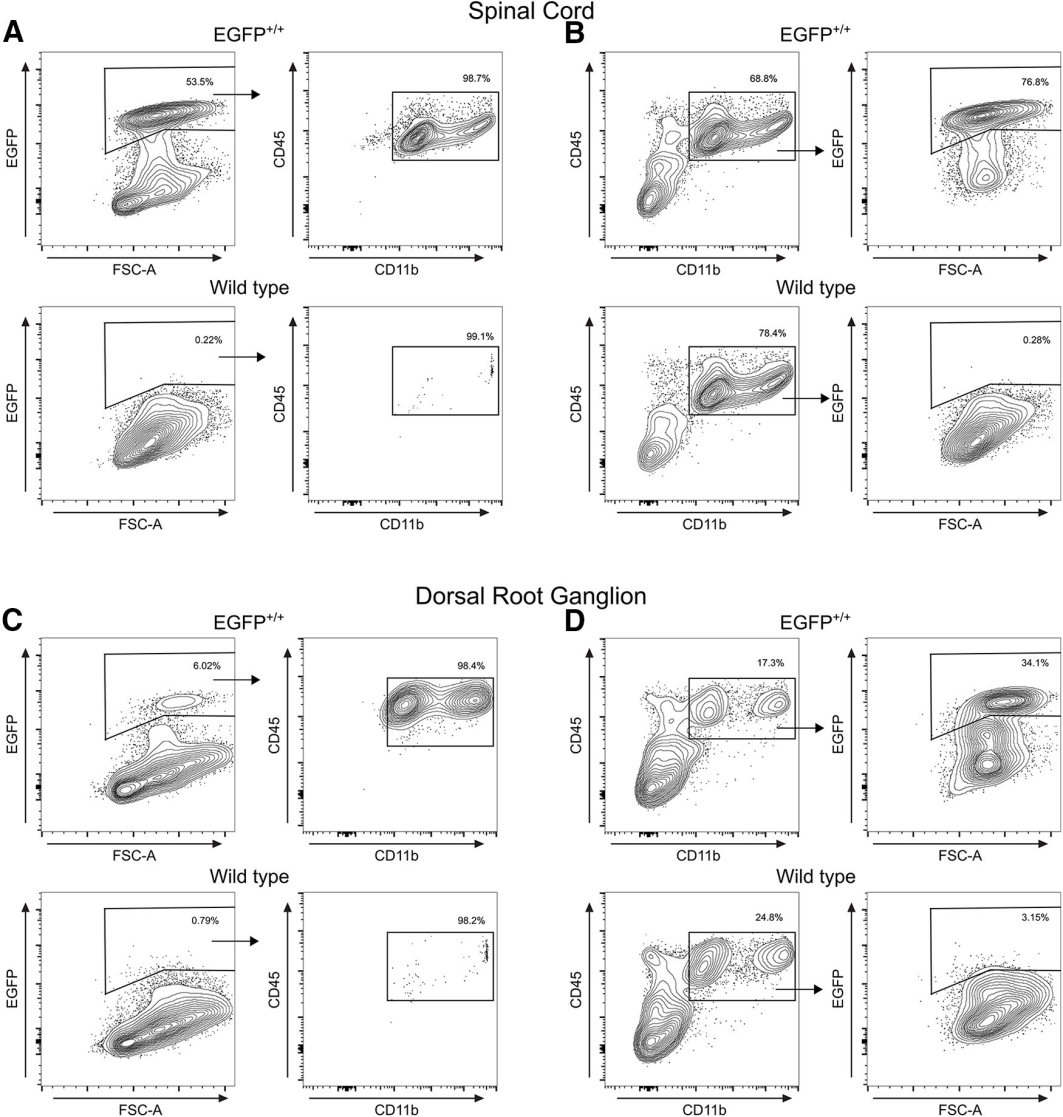
Comparison of EGFP signal in spinal cord and dorsal root ganglion. ***A***, Spinal cord cells isolated from either EGFP^+/+^ (top) or wild-type littermates (bottom) at P7 were gated as EGFP^+^ (left) and then gated as CD11b^+^/CD45^+^ (right). ***B***, The same samples from ***A*** were reanalyzed, gated as CD11b^+^/CD45^+^ (left), then gated for EGFP expression (right). ***C***, Dorsal root ganglion cells isolated from either EGFP^+/+^ (top) or wild-type littermates (bottom) at P7 were gated as EGFP^+^ (left) and then gated as CD11b^+^/CD45^+^ (right). ***D***, The same samples from ***C*** were reanalyzed, gated as CD11b^+^/CD45^+^ (left), then gated for EGFP expression (right). Contour lines in ***A–C*** represent 95% of the data at 5% intervals. Black boxes in ***A–D*** indicate gates, and the percentage of cells within each gate are shown.

Next, we examined EGFP expression in the spinal cord and dorsal root ganglion to determine the extent of EGFP expression in other neural tissues. We used flow cytometry to analyze the expression of CD45 and CD11b of acutely isolated cells from the spinal cord (Fig. 4*A*,*B*) and dorsal root ganglion (Fig. 4*C*,*D*) of P7 EGFP^+/+^ or wild-type littermates. In the spinal cord, ∼59.8% of cells were EGFP^+^ from EGFP^+/+^ animals compared with 0.2% of cells from wild-type animals (Tables 2, 4). Of the EGFP^+^ cells, 99.2% were identified as CD11b^+^/CD45^+^ (Fig. 4*A*, Table 3). In the dorsal root ganglion, 6.2% of cells were EGFP^+^ in EGFP^+/+^ animals (compared with 0.8% in wild-type animals), of which 97.6% were CD11b^+^/CD45^+^ (Fig. 4*C*, Table 4).

**Table 4:** Iba1-EGFP Spinal cord and dorsal root ganglion flow cytometry results

	Wild type	EGFP^+/+^	Figure reference
PN7 spinal cord	(*n* = 1)	(*n* = 2)	
EGFP^+^ cells (mean counts ± SD, % live singlets)	112, 0.2%	35,385 ± 3896, 59.8 ± 8.8%	Figure 4*A*
Subgate: proportion of EGFP^+^ cells that are CD11b^+^/CD45^+^ (counts ± SD, % EGFP^+^ ± % SD)	N/A	35,105 ± 4094, 99.15 ± 0.6%	
CD11b^+^/CD45^+^ cells (counts/live singlets, % live singlets)	40163, 78.4%	43,770 ± 2592, 73.9 ± 7.1%	Figure 4*B*
Subgate: proportion of CD11b^+^/CD45^+^ cells that are EGFP^+^ (counts ± SD, % CD11b^+^/CD45^+^ ± % SD)	111, 0.3%	35,755 ± 4207, 80 ± 4.6%	
PN7 dorsal root ganglion	(*n* = 1)	(*n* = 3)	
EGFP^+^ cells (mean counts ± SD, % live singlets)	170, 0.8%	2350 ± 240, 6.18 ± 2.0%	Figure 4*C*
Subgate: proportion of EGFP^+^ cells that are CD11b^+^/ CD45^+^ (counts ± SD, % EGFP^+^ ± % SD)	N/A	2292 ± 224, 97.6 ± 0.7%	
CD11b^+^/CD45^+^ cells (counts/live singlets, % live singlets)	5308, 24.8%	7162 ± 993, 18.3 ± 2.7%	Figure 4*D*
Subgate: proportion of CD11b^+^/CD45^+^ cells that are EGFP^+^ (counts ± SD, % CD11b^+^/CD45^+^ ± % SD)	167, 3.2%	2289 ± 226, 32.4 ± 6.1%	

We again analyzed the data in reverse, first gating for CD11b^+^/CD45^+^ myeloid cells and then by EGFP. In the spinal cord, 73.9% of cells were CD11b^+^/CD45^+^ and 80.0% of those cells were EGFP^+^ (Fig. 4*B*, Table 4), while 18.3% of cells in the dorsal root ganglion were CD11b^+^/CD45^+^ with 32.4% of those cells being EGFP^+^ (Fig. 4*D*, Table 4).

**Figure 5.**
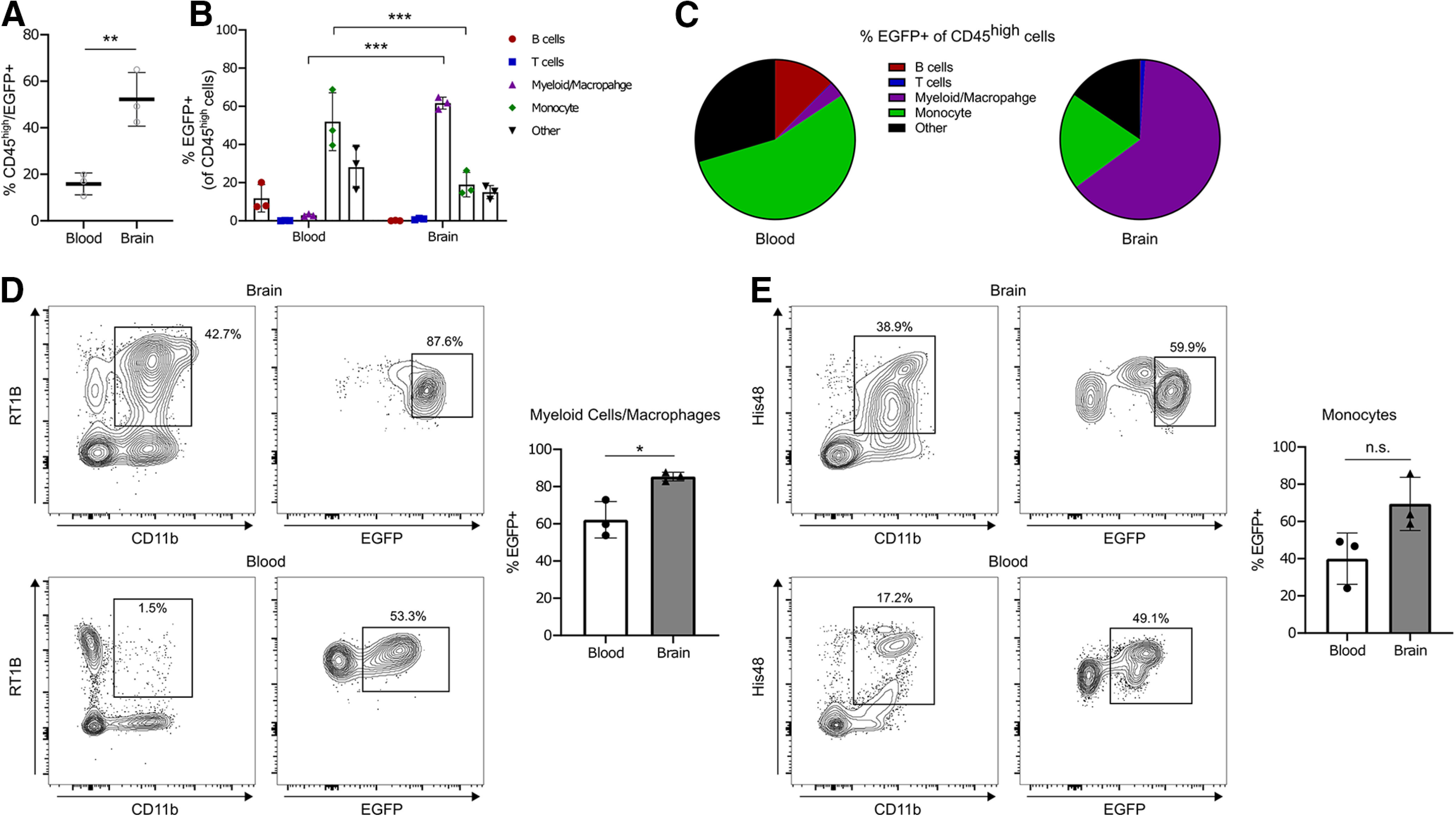
Flow cytometric characterization of EGFP^+^ peripheral cells in the adult. ***A***, Quantification of the percentage of CD45^high^ peripheral immune cells expressing EGFP in the brain compared with the blood. ***B***, Graph depicting the population subsets of CD45^high^/EGFP^+^ cells in the brain and blood. (B cells = CD45^high^, CD45R^+^; T cells = CD45^high^, CD3^+^; myeloid cells/macrophages = CD45^high^, CD11b^+^, RT1B^+^; monocytes = CD45^high^, CD11b^+^, His48^+^; other = CD45^high^, additional immune cells not determined by this panel). ***C***, Pie charts showing the relative distribution of CD45^high^/EGFP^+^ immune cells in the blood (left) and brain (right). ***D***, Contour plots depicting the gating strategy for myeloid cells/macrophages in the brain (top) and blood (bottom). Quantification of the percentage of myeloid cells/macrophages expressing EGFP in the blood and brain. ***E***, Contour plots showing the gating strategy for monocytes in the brain (top) and blood (bottom). Quantification of the percentage of monocytes expressing EGFP in the blood and brain. Contour lines (***D***, ***E***) represent 95% of the data at 5% intervals. Bars represent the mean ± SD. Open circles and solid shapes represent individual animal datapoints. *p < 0.05; **p < 0.01; ***p < 0.001.

To further characterize the CD45^high^ hematopoietic cells (i.e., nonmicroglia cells) that express EGFP, we performed flow cytometry to analyze blood and brain samples from adults. In the brain, 52.2% of CD45^high^ cells were EGFP^+^, while in the blood only 15.9% of CD45^high^ cells were EGFP^+^ (*p *=* *0.0072^m^; Fig. 5*A*). Other than microglia, myeloid cells/macrophages (CD45^high^/CD11b^+^/RT1B^+^) were the most prevalent EGFP-expressing cell type in the brain, comprising 61.7% of EGFP^+^ cells compared with 2.9% in the blood (*p *<* *0.001^p^; Fig. 5*B*,*C*) and were consistent with known Iba1 expression in myeloid cells (Imai and Kohsaka, 2002; Ji et al., 2007; Jeong et al., 2013). Moreover, 85.4% of all myeloid cells/macrophages were EGFP^+^ in the brain, while 62.2% were EGFP^+^ in the blood (*p *=* *0.016^s^; Fig. 5*D*). In the blood, EGFP^+^ cells were mostly monocytes (CD45^high^/CD11b^+^/His48^+^; 52.0%) and were much less prevalent in the brain (19.0%, *p *=* *0.025^q^; Fig. 5*B*,*C*); of the total monocyte population, 40% were EGFP^+^ in the blood and 69.4% were EGFP^+^ in the brain (*p *=* *0.062^t^; Fig. 5*E*). Both T cells (0.1% in blood vs 1.0% in brain^o^) and B cells (11.8% in blood vs 0.1% in brain^n^) were far less frequent in the EGFP^+^ population of both tissue types. Finally, our antibody panel failed to label a population of EGFP^+^ cells (termed “other”; Fig. 5*B*,*C*) that represented 28.1% of cells in the blood and 15.0% in the brain^r^. Given the design of our antibody panel, these cells are most likely nonclassical monocytes as His48 mostly labels classical monocytes and neutrophils, and that would explain the increased prevalence of this population in the blood compared with the brain (Barnett-Vanes et al., 2016).

### Histologic validation of EGFP^+^ cells

**Figure 6.**
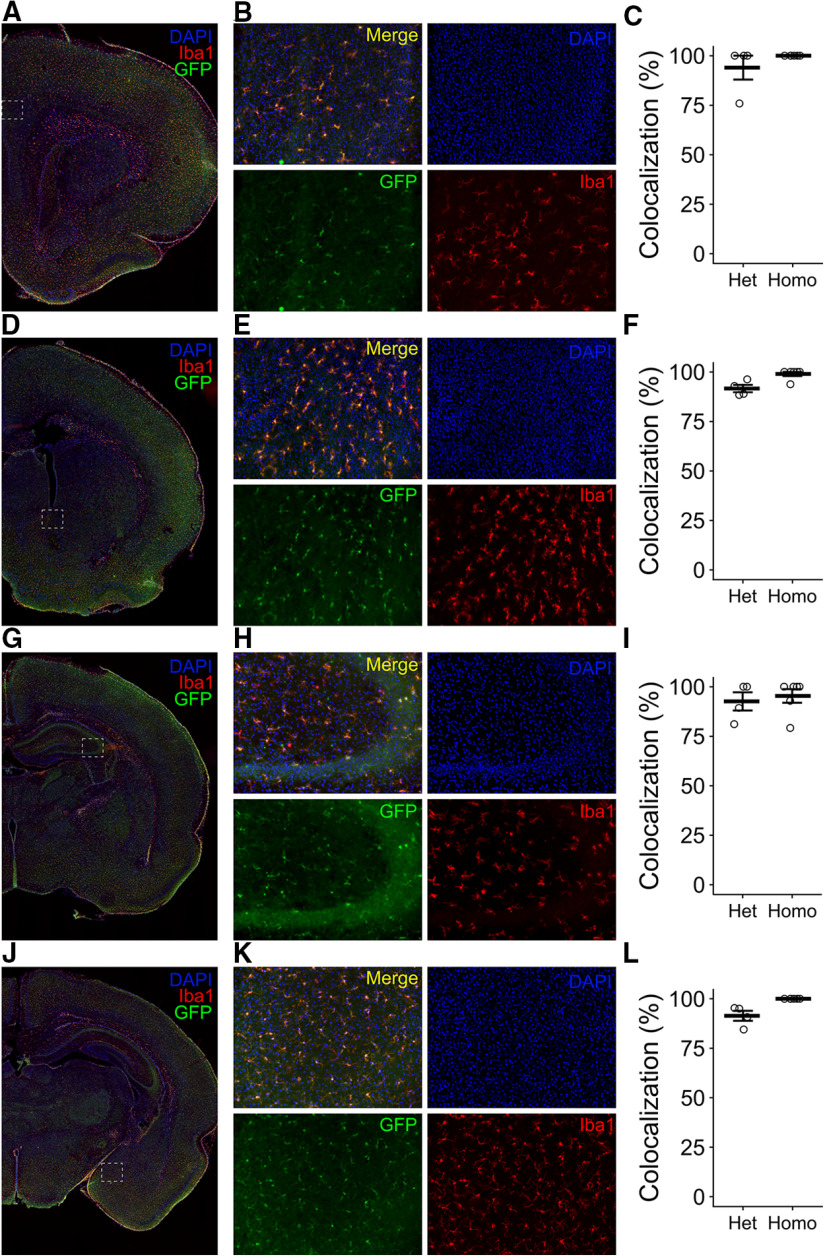
Histologic validation of knock-in efficiency. ***A***, ***D***, ***G***, ***J***, Representative coronal images from EGFP^+/−^ animals antibody labeled for Iba1 (red), GFP (green), and colabeled with DAPI (blue). White box shows the region analyzed for the prefrontal cortex (***A–C***), nucleus accumbens (***D–F***), hippocampus (***G–I***), and amygdala (***J–L***). ***B***, ***E***, ***H***, ***K***, Representative single-channel and merged images at 20× magnification. ***C***, ***F***, ***I***, ***L***, Quantification of the percentage of colocalization between GFP and Iba1 in heterozygous and homozygous animals. Bars represent the mean ± SEM. Open circles represent individual animal datapoints.

To determine whether EGFP expression could be reliably detected in all microglia throughout the brain, we used histology to assess the colocalization of Iba1 and GFP by immunolabeling for both proteins. We quantified microglia in several regions on P7, as follows: the prefrontal cortex (PFC; Fig. 6*A–C*), nucleus accumbens (NAc; Fig. 6*D–F*), hippocampus (Hipp; Fig. 6*G–I*), and amygdala (Amyg; Fig. 6*J–L*). We found that across all regions, >90% of all Iba1^+^ cells colabeled as GFP^+^ in EGFP^+/−^ animals (93.97% in PFC^u^, 91.59% in NAc^v^, 92.59% in Hipp^w^, 91.39% in Amyg^x^) and ∼98% of Iba1^+^ cells were GFP^+^ in EGFP^+/+^ animals (100% in PFC^u^; 98.96% in NAc^v^; 95.34% in Hipp^w^; 100% in Amyg^x^).

### *Ex vivo* and *in vivo* imaging of EGFP^+^ cells

**Figure 7.**
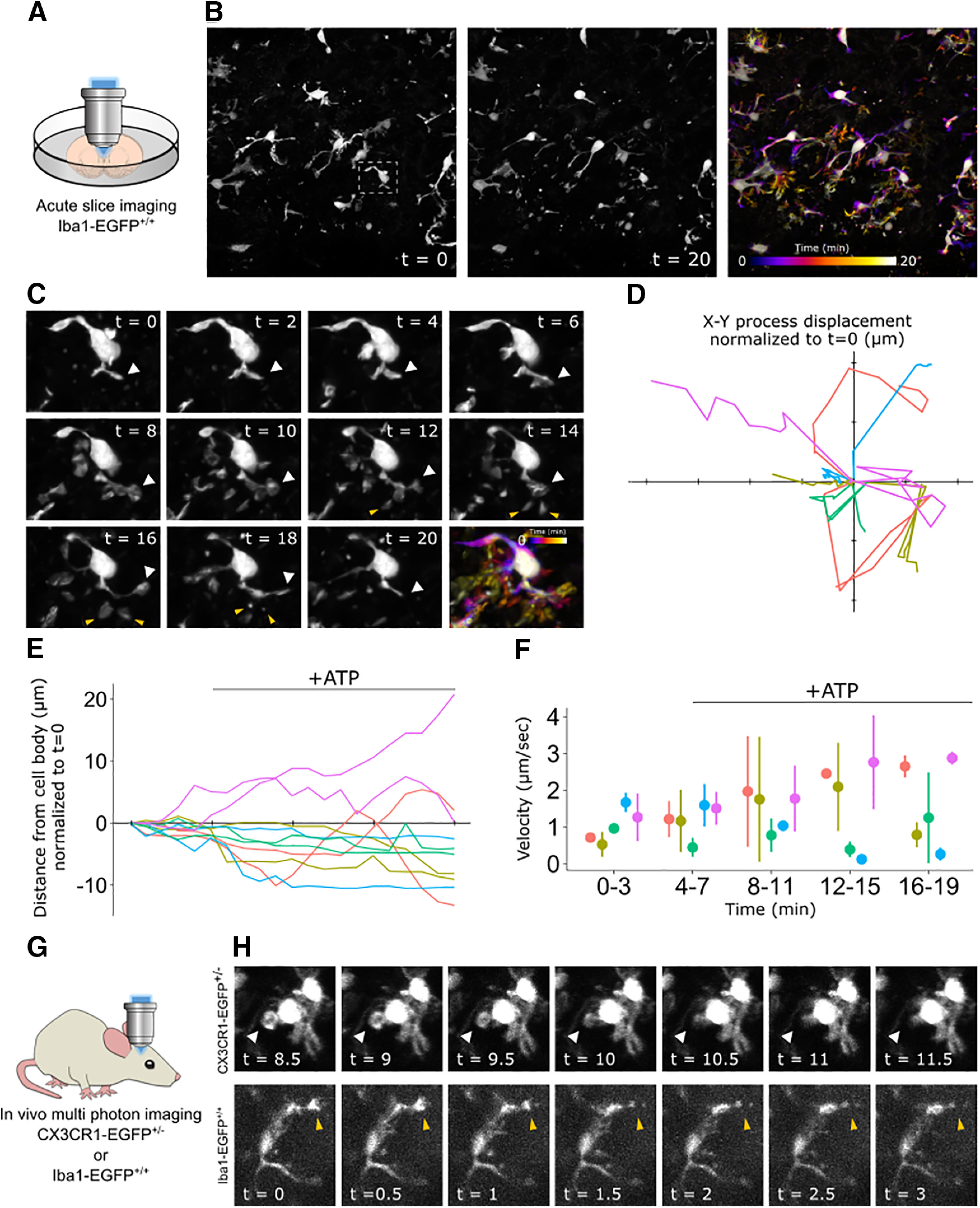
*Ex vivo* and *in vivo* time-lapse imaging of EGFP^+^ microglia. ***A***, Experimental setup; an acute brain slice was prepared from an Iba1-EGFP^+/+^ rat pup and placed under a confocal microscope for imaging. ***B***, Representative field of view of microglia from an EGFP^+/+^ animal at the start (0 min; left) and end (20 min; middle) of the assay. The same field of view is pseudocolored to represent the microglia position throughout the duration of the assay (right). The white box shows the cell represented in ***C***. ***C***, Depiction of a single microglia at 2 min intervals throughout the assay with the corresponding time pseudocolor image. White arrows track a single major process through time. Yellow arrows indicate process bifurcations that extend and retract in response to ATP application, which was bath applied at a concentration of 1 mm in aCSF after 4 min of baseline imaging. ***D***, Quantification of microglia process displacement throughout the duration of the assay. The *x*- and *y*-axes represent the *x*- and *y*-coordinates relative to the microscope image. Individual traces show process movement from *t* = 0 (*x*/*y* origin) to *t* = 20. Axis tick marks represent 5 μm. ***E***, Quantification of the microglia process retraction and extension throughout the duration of the assay. The *x*-axis tick marks represent 5 min intervals. ***F***, Quantification of the microglia process velocity throughout the duration of the assay. ***G***, Experimental setup; an anesthetized *Iba1-EGFP^+/+^* rat or *CX3CR1-GFP^+/−^* mouse was head fixed for two-photon imaging through the skull. ***H***, Depiction of a single microglia from a *CX3CR1-EGFP^+/−^* mouse (top) and an *Ib1-EGFP^+/+^* rat (bottom) imaged *in vivo* with two-photon microscopy at ∼30 s intervals. White arrows track the internalization of a phagocytic cup and yellow arrows track the retraction of a microglial process. In ***C***, ***D***, and ***E***, each color represents a different cell; same color traces represent different processes from the same cell. In ***D*** and ***E***, black bar indicates the time and duration of ATP application. In ***F***, data are represented as the mean ± range.

One of the most significant advantages of microglia reporter mice has been the ability to monitor microglia dynamics in real time. To determine whether the Iba1-EGFP rat was suitable for live imaging studies, we first used confocal microscopy to image EGFP^+^ cells in acute brain slices from P7 EGFP^+/+^ animals. We imaged for 4 min to measure microglia dynamics under baseline conditions, then applied 1 mm ATP to the bath solution for the remainder of the 20 min assay. The EGFP signal was sufficient to image multiple cells across a large field of view and track microglial process dynamics over time (Fig. 7*A*,*B*, Movie 1). Moreover, we were able to identify filopodia formation in response to ATP application (Fig. 7*C*) and quantify measures of microglia movement such as *x–y* displacement (Fig. 7*D*), process retraction/extension (Fig. 7*E*), and process velocity (Fig. 7*F*).

Movie 1. Time-lapse video of EGFP^+^ microglia in acute slices. Representative field of view of microglia from an EGFP^+/+^ animal. Confocal *z*-stacks were acquired in 1 min intervals. ATP (1 mm in aCSF) was bath applied after the fourth frame, as denoted by “+ATP” in the video. Scale bar, 20 μm. 10.1523/ENEURO.0026-21.2021.video.1

To determine whether this model is equally suitable for *in vivo* studies, we used multiphoton imaging to visualize microglia dynamics through a thinned skull preparation in P14 *Iba1-EGFP^+/+^* rats. In parallel and as a positive control, we used the same procedure to image adult *CX3CR1-EGFP^+/−^* mice, which are widely used to study microglia *in vivo* (Jung et al., 2000; Guttenplan and Liddelow, 2019; Moseman et al., 2020). Though *CX3CR1-EGFP^+/−^* microglia exhibited brighter fluorescence, *Iba1-EGFP^+/+^* microglia were dynamic from frame to frame (Fig. 7*H*), matching the activity observed in the *CX3CR1-EGFP* mice.

## Discussion

Described here is a novel transgenic rat, *SD-Tg(Iba1-EGFP)Mmmc*, that leverages EGFP expression under the control of the *Iba1* promoter. Utilization of a 2A self-splicing peptide as a linker between the *EGFP* and *Iba1* coding sequence expressed in series from the same gene locus enables the expression of EGFP with the same specificity, timing, and abundance of endogenous Iba1. Cleavage at the P2A peptide in particular has been shown to be highly efficient, both *in vitro* and *in vivo*, and in mice <10% of translational products are uncleaved fusion protein (Kim et al., 2011; Liu et al., 2017). Our *SD-Tg(Iba1-EGFP)Mmmc* rats show the same high-efficiency splicing, as we were unable to detect any EGFP-P2A-Iba1 fusion protein using Western blotting. This system also preserves endogenous levels of Iba1 protein in our transgenic rats. Using a combination of flow cytometry, PCR, and immunohistochemistry, we show that EGFP expression faithfully identifies microglia in the brain. We further demonstrate the utility of this model in expanding the repertoire of microglia techniques in the rat, by live imaging microglia process motility in acute slice and *in vivo* preparations.

Iba1 protein, while traditionally held as the “gold standard” for identifying microglia in the brain, is also expressed by a variety of monocytes/macrophages throughout the body and is robustly expressed in the spermatids of the testis (Köhler, 2007). While we did not perform a comprehensive analysis across tissue types, in the brains of our transgenic model EGFP expression was almost entirely limited to microglia as >95% of EGFP^+^ cells colabeled as CD11b^+^/CD45^int^ by flow cytometry and between 95% and 100% of EGFP^+^ cells colabeled as Iba1^+^ by histology depending on genotype. Moreover, EGFP expression was robust in both neonates and adults, demonstrating that the transgene is expressed early and is stable into early adulthood. By using flow cytometry, we detected EGFP^+^ cells in the blood and a small population of EGFP^+^ nonmicroglial cells in the brain. EGFP^+^ cells in the blood were largely monocytes, while nonmicroglial EGFP^+^ cells in the brain were largely myeloid cells/macrophages. A significantly larger percentage of myeloid cells/macrophages expressed EGFP in the brain compared with the blood, which is consistent with an upregulation of Iba1 in myeloid cells on entering the brain and establishing tissue residence (Imai and Kohsaka, 2002; Kishimoto et al., 2019; Swanson et al., 2020). Moreover, the percentage of monocytes expressing EGFP was not different between the blood and brain, which is expected in a naive, nonactivated state where circulating monocytes are not actively recruited to the brain.

Outside of the brain, we were able to detect EGFP expression in other neural tissues, for example the spinal cord and dorsal root ganglion. The spinal cord was enriched with EGFP^+^ cells, whereas the dorsal root ganglion had far fewer EGFP^+^ cells, which is to be expected given the distributions of microglia and macrophages in CNS and peripheral nervous system tissue, respectively (Xuan et al., 2019; Kolter et al., 2020). In both tissues, the vast majority of EGFP^+^ cells were CD11b^+^/CD45^+^, further demonstrating that EGFP expression is highly specific to select immune cell populations.

The *SD-Tg(Iba1-EGFP)* rat that we describe here presents several significant advancements over traditional methods. First, endogenous fluorescent reporters greatly facilitate the analysis and purification of Iba1^+^ cells, without the need for multiple antibody-labeling steps or complicated antibody panels. By minimizing tissue processing, microglia can be quickly isolated from the brain and analyzed by flow cytometry or FACS, and from much smaller starting volumes or brain regions. The ability to use FACS to sort and then sequence single-cell RNA using microglia from genetically modified mice expressing EGFP has considerably expanded our understanding of these critical cells in the mature and developing brain and in response to disease or injury (Hammond et al., 2019; Li et al., 2019). To date, this level of analysis has been out of reach for researchers using the rat model.

Second, endogenous reporters drastically improve the quality and capabilities of both live and *ex vivo* imaging studies. Compared with previous methodology, which largely relied on the application of fluorescent labels that bind to microglia membrane receptors (e.g., ib4 labeling), the GFP signal in this rat acts as a cell fill, which allows detailed imaging of microglia processes and dynamics over time. This can be exploited for multiple purposes, including imaging of microglia motility in acute slices, as was done here. There is also the potential for *in vivo* imaging for longitudinal studies of particular brain regions after stroke, traumatic brain injury (TBI), or other manipulations, as was done in the mouse (Pernici et al., 2019), and is now feasible in the rat, as demonstrated here. Given the importance of microglia function during early brain development (Frost and Schafer, 2016), the larger size of rats may allow for *in vivo* imaging during developmental ages that would not be feasible in mice. Last, the celebrated and impactful observations that microglia can engulf microstructures such as synapses and thereby prune them to sculpt neural circuit development, was achieved with the use of a mouse model in which microglia expressed GFP (Schafer and Stevens, 2013). This transformative finding can now be corroborated and expanded on in the rat model.

Third, given the fidelity of EGFP expression in blood monocytes and macrophages, this rat may be beneficial for studying immune responses in models of ischemic stroke, infection, or TBI where peripheral monocytes infiltrate the brain (Yang et al., 2019). Iba1 is a marker of peripheral tissue-resident macrophages, so using this rat may be useful in studying an array of other myeloid cell-driven responses throughout the body (Wijesundera et al., 2013; Suenaga et al., 2016; Ma et al., 2017). A further hidden benefit of this feature is the ability to screen animals for EGFP expression in the brain by first screening the blood. Although brain EGFP expression was higher than that in blood, the blood CD45 compartment still showed a one-log shift in EGFP expression, indicating a potential for flow cytometric analysis of the blood as a screening tool for transgene positivity. Our recommended panel for blood screening (Fig. 3*A*,*D*) would include only a CD45 antibody and require analysis of only two fluorescent channels, making this screening adaptable to most, if not all, flow cytometers. We found that at P7 and in adults, Iba1-EGFP^+^ cells are present in the blood and comparable to expression in the brain.

In summary, we report here the generation of a novel transgenic rat, *SD-Tg(Iba1-EGFP)Mmmc*, in which microglia and some peripheral immune cells express EGFP protein in sufficient quantity to allow for flow sorting and FACS, and *in vivo* and *ex vivo* visualization. The rat has long been a favored animal model in neuroscience, but the inability to readily genetically modify them has diminished use over the past 2 decades. With the advent of CRISPR technology, this is likely to change, and we offer this model as one such example of the changes to come.

## Synthesis

Reviewing Editor: Lindsay De Biase, University of California, Los Angeles Decisions are customarily a result of the Reviewing Editor and the peer reviewers coming together and discussing their recommendations until a consensus is reached. When revisions are invited, a fact-based synthesis statement explaining their decision and outlining what is needed to prepare a revision will be listed below. The following reviewer(s) agreed to reveal their identity: Amit Agarwal.

In this manuscript, the authors describe generation of a new transgenic Sprague-Dawley rat line expressing EGFP under the control of endogenous Iba1/Aif1 promoter. The authors used CRISPR/Cas9 technology to knock-in EGFP into the Iba1 gene locus. Using flow cytometry, immunohistochemistry, and quantitative real-time PCR based gene expression analysis, the authors show that in the brain, EGFP is expressed mainly by microglial cells, and EGFP expression starts perinatally and persists in adulthood. Additionally, the authors performed flow cytometry analysis on blood and found a sub-population of macrophages and monocytes express EGFP in their transgenic rats.

Both reviewers agreed that this tool has excellent potential to advance research in the microglial field. However, to capture this new rat line’s full potential and to ensure its usability as a versatile tool for neuroimmunological research, they need to provide more data related to the generation, characterization, and potential utility of the rat line.

Major concerns:

1) This manuscript reported the generation of a new transgenic rat line to specifically label microglia cells in the rat nervous system. However, the authors don’t provide sufficient details of how these transgenic rats were generated. The results section needs to refer to and describe Figure 1. The authors should elaborate on their CRISPR-Cas9 strategy, including the location of the guide-RNA used for targeting EGFP in the Iba1 locus. The authors should also provide the details of high scoring off-target genes that can be targeted by gRNAs used and confirmation that these gene loci are intact in their transgenic rat line. From the cartoon depicting the gene targeting strategy in Figure 1a, it seems due to the usage of P2A peptide, the endogenous expression of Iba1 protein might be preserved. However, the authors didn’t provide any information on this. It is essential to clarify (a) what proportion of EGFP is expressed as a fusion protein with Iba1 and (b) if Iba1 protein is expressed at the endogenous levels in heterozygote or homozygote transgenic rats?

2) Iba1-EGFP rats have the potential to become a widely used tool to study microglial cells in the nervous system. However, the authors exclusively focused their analysis on the brain. Since this study reports the generation of a transgenic tool, it is necessary to include the expression analysis (at least histological analysis) for other central and peripheral nervous system tissue such as spinal cord and dorsal root ganglion.

3) The authors do not sufficiently argue for a rationale of using rats over mice. What precisely is this going to allow the field to do now that it can’t already do in mice? There is a single statement to this effect in the last paragraph of the Discussion and some cursory descriptions in the introduction. But the authors should devote a paragraph to the strength of the rat model and thus a need for this mouse. This would fit very well in the last paragraph of the Introduction where “the advantages of using a rat model” is mentioned but not detailed and could be reiterated and expanded (if needed) in the discussion. For example, the authors could describe the richer learning and behavioral repertoire of rats and what might be learned by FACS sorting or imaging microglia in the context of these more complex behavioral paradigms. The authors could also discuss potential for neonatal in vivo imaging as rat brains, being larger in size, would facilitate in vivo surgeries for in vivo imaging. Spinal cord imaging may similarly be more tractable with the larger size of the spinal cord. For applications that are limited by collection of a sufficient number of microglia (proteomics?), the rat model may facilitate analysis of sufficient microglia from discrete brain regions that would be too small to analyze from mice.

4) Although Figure 6 presents the proof-of-principle for these rats’ usability for live microglia imaging, the live imaging analysis is very cursory. Although it would be ideal to demonstrate that some form of in vivo imaging that is not currently possible in mice (or is very difficult in mice) becomes possible in rats, we realize this is beyond the scope of the current manuscript. However, the authors should aim to be more thorough in their acute brain slice imaging characterization and quantification to show that this is at least as good as what is done in mice and, hence, there is good reason to be confident that this tool will work well for challenging in vivo imaging applications. The authors should show quantification and required statistical tests across multiple experiments. They should consider applying a laser lesion or pipette source of ATP to demonstrate targeted process outgrowth toward a focal injury or point source of ATP, which is a critical metric of microglial motility in vivo and in acute brain sections in mice. Theoretically, it would be possible to accomplish acute brain slice imaging of microglia in rats using fluorescent isolectins (Madry et al. 2018). Is the EGFP signal within microglia in these rats superior to this approach? One limitation for in vivo imaging of microglia in some mouse models has been the signal intensity from the fluorophore. If possible, the authors should show whether the EGFP signal in these rats is bright enough to resolve the finest small filopodia of microglia (Bernier et al 2019), suggesting that it will work well for high level in vivo imaging applications (although this would likely require multiphoton in slice imaging approaches).

Minor Concerns

1) The numbers in the text for the % of cells obtained by flow cytometry don’t seem to match up with the numbers in the Figures proper e.g. 9.8% in the text of Fig. 2A but 8.5% in the Figure proper e.t.c. This seems to bee the case in several Figure panels. The authors should check to make sure that these numbers are congruent.

2) Several places through the text, the authors although looking only at P60 young adults, use the phrase “throughout life”. While this is likely true, the authors do not provide evidence for this. Therefore these should be modified to state “into early adulthood” e.g. second paragraph of the Results, the end of the paragraph on “Histological validation of EGFP+ cells” and the second paragraph of the Discussion. The authors could state “into early adulthood and may do so throughout life”.

3) Although the authors use Iba1 and CX3CR1 (third paragraph of the Results) as “microglia-enriched genes", these genes can be expressed by other border-associated brain macrophages like perivascular macrophages. It would be nice if the authors can also sort or do IHC using P2RY12 which is a more selective microglial marker not expressed by perivascular macrophages (Goldman et al., 2016).

4) In the fourth paragraph of the discussion, the authors mention the need for GRIIN lenses for in vivo imaging of microglia. This is not NEECESSARILY the case. Simple cortical imaging does not require GRIN lenses for two photon imaging. The authors should revise this statement.

5) In the penultimate sentence of 5th paragraph of Discussion the authors state “...making this screening adaptable to most all flow cytometers”. This would better read “”...making this screening adaptable to most (IF NOT ALL) flow cytometers.

6) The legend of the video fails to detail the application of ATP which it should include.

7) In table 2 (adult), it is unexpected that 37% of all brain cells are EGFP/+; this number should be around 10% (i.e., the proportion of microglia in the adult brain)? Also, there seemed to be an increased number of CD11b+/CD45int cells in Iba1-EGFP rats (19% in control vs. 36% in the transgenic rats). Does this indicate ongoing inflammation or resident cell-types in the brain other than microglia or infiltrating cells from the periphery express EGFP? Instead of such a confusing table, it will be intuitive and helpful for readers to see the plots with the individual data points in the figures, and the exact number can be presented in the text of the manuscript.

8) The authors should provide FACS scatter plots from the matching control groups (Fig. 3A and D), especially when the authors discuss about a distinct population of CD45/EGFP+ cells in transgenic vs. control rats.

9) Figure 4B indicates that of all EGFP+ cells in the brain, 60% are microglial cells (myeloid/macrophage origin), and the rest 40% are monocytes and other cells. How do these numbers corroborate with the distinct cell-types in the brain expressing Iba1? In general, the information provided in Figure 4 is confusing and requires a table or pie chart to explain which ’immune’ cell-types are there in the adult rat brain and what proportion of these are EGFP+. It is essential to precisely know the identity of all cell-types being sorted when Iba1-EGFP rats are used.

10) Throughout the study, the authors used either homozygote or heterozygote rats for experiments. For all experiments, please provide the genotype of transgenic and control rats in all the figures.

11) The authors mentioned 94.9% of EGFP+ cells were CD11b+/CD45int in Iba1-EGFP rats; later, they refer that myeloid cells/macrophages (CD45high/CD11b+/RT1B+) were the most prevalent cell-type in the brain, comprising 61.7% of EGFP+ cells. Such comparisons are quite confusing and the authors need to clarify this in the text.

## Author Response

Dear Dr. De Biase,

Thank you for the opportunity to provide a revised version of our manuscript eN-MNT-0026-21, Generation of an Iba1-EGFP transgenic rat for the study of microglia in an outbred rodent strain. We have conducted additional experiments, edited and revised the manuscript and figures and find it greatly improved in response to the many helpful comments and concerns expressed by the reviewers. Below please find a point-by-point response to the reviewers with their remarks in BLACK and our responses in BLUE. To facilitate the review of the revised version of the manuscript, any additions and edits are in BLUE.

Response to reviewers: Major concerns:

1) This manuscript reported the generation of a new transgenic rat line to specifically label microglia

cells in the rat nervous system. However, the authors don’t provide sufficient details of how these transgenic rats were generated. The results section needs to refer to and describe Figure 1. The authors should elaborate on their CRISPR-Cas9 strategy, including the location of the guide-RNA used for targeting EGFP in the Iba1 locus. The authors should also provide the details of high scoring off-target genes that can be targeted by gRNAs used and confirmation that these gene loci are intact in their transgenic rat line.

-We have included the sequence and location of the guideRNA used to target the transgene insertion in the Material and Methods section of our revision. Although the company we contracted to generate this transgenic rat provided us with sequence information for the guide RNA that was ultimately used, we were not provided with information about potential off-target sites for this guide RNA. Because Applied StemCell Inc. was contracted for their expertise in design, generation and validation of rat CRISPR models, validation of transgene targeting was left in their hands. We have also described the transgene construct, subsequent genotyping of of the SD-Tg (Iba1-EGFP)Mmmc rats, and validation and characterization of EGFP and Iba1 expression in these rats in the Results section of our revision, as referred to in Figure 1.

From the cartoon depicting the gene targeting strategy in Figure 1a, it seems due to the usage of P2A peptide, the endogenous expression of Iba1 protein might be preserved. However, the authors didn’t provide any information on this.

-This reviewer is correct, use of the P2A linker peptide provides bicistronic expression of EGFP and Iba1

from the transgene, ensuring that unmodified Iba1 is present at endogenous levels regardless of genotype. We have now included this information in the Results section (Figure 1) of our revision.

It is essential to clarify (a) what proportion of EGFP is expressed as a fusion protein with Iba1

-Determination of the proportion of uncleaved fusion protein can be done precisely using mass spec or via estimation using Western blot. Unfortunately, we did not have access to mass spec facilities for these studies. However, we have included Western blot data for EGFP protein in the cortex of EGFP-/- and EGFP+/+ rats. As shown in Figure 1C, anti-GFP antibody detects a band of approximately 35 kDa, which corresponds to EGFP protein with the 17 amino acid P2A peptide on the C-terminus, in cortical tissue from EGFP+/+ but not EGFP-/- rats. Higher molecular weight bands corresponding to a EGFP-P2A- Iba1 fusion protein, which is predicted at approximately 52 kDa, were not detected. Because of the imperfect sensitivity of Western blotting using tissue homogenates, we recognize that this does not prove a 0% proportion of EGFP-P2A-Iba1 fusion protein. However, this does indicate that the amount of

fusion protein, if any, is a very small percentage of the transgene product. This is in line with the >90% cleavage efficiency of the P2A peptide in zebrafish, mouse, and mammalian cell lines as reported in the literature (Kim et al., 2011; Liu et al., 2017).

(b) if Iba1 protein is expressed at the endogenous levels in heterozygote or homozygote transgenic rats? -We agree that it is important to demonstrate whether the transgene alters levels of Iba1 protein. To address this, we have included a Western blot quantification of Iba1 protein in the cortex of EGFP-/-, EGFP+/- and EGFP+/+ rats at P18. These data demonstrate that there is no difference among genotypes in the amount of Iba1 protein of the expected molecular weight (ie, not as a fusion protein with EGFP) (Figure 1D).

2) Iba1-EGFP rats have the potential to become a widely used tool to study microglial cells in the nervous system. However, the authors exclusively focused their analysis on the brain. Since this study reports the generation of a transgenic tool, it is necessary to include the expression analysis (at least histological analysis) for other central and peripheral nervous system tissue such as spinal cord and dorsal root ganglion.

-We appreciate the feedback and have included a new flow cytometry analysis of EGFP expression in the spinal cord and dorsal root ganglion (Figure 4 and Table 3).

3) The authors do not sufficiently argue for a rationale of using rats over mice. What precisely is this going to allow the field to do now that it can’t already do in mice? There is a single statement to this effect in the last paragraph of the Discussion and some cursory descriptions in the introduction. But the authors should devote a paragraph to the strength of the rat model and thus a need for this mouse. This would fit very well in the last paragraph of the Introduction where “the advantages of using a rat model” is mentioned but not detailed and could be reiterated and expanded (if needed) in the discussion. For example, the authors could describe the richer learning and behavioral repertoire of rats and what

might be learned by FACS sorting or imaging microglia in the context of these more complex behavioral paradigms. The authors could also discuss potential for neonatal in vivo imaging as rat brains, being larger in size, would facilitate in vivo surgeries for in vivo imaging. Spinal cord imaging may similarly be more tractable with the larger size of the spinal cord. For applications that are limited by collection of a sufficient number of microglia (proteomics?), the rat model may facilitate analysis of sufficient microglia from discrete brain regions that would be too small to analyze from mice.

-As requested, we have expanded the introduction and discussion to include a dedicated paragraph that highlights the advantages of a rat model and expands upon the possible use of this rat model for in vivo imaging applications.

4) Although Figure 6 presents the proof-of-principle for these rats’ usability for live microglia imaging, the live imaging analysis is very cursory. Although it would be ideal to demonstrate that some form of in vivo imaging that is not currently possible in mice (or is very difficult in mice) becomes possible in rats, we realize this is beyond the scope of the current manuscript. However, the authors should aim to be more thorough in their acute brain slice imaging characterization and quantification to show that this is at least as good as what is done in mice and, hence, there is good reason to be confident that this tool will work well for challenging in vivo imaging applications. The authors should show quantification and required statistical tests across multiple experiments. They should consider applying a laser lesion or pipette source of ATP to demonstrate targeted process outgrowth toward a focal injury or point source of ATP, which is a critical metric of microglial motility in vivo and in acute brain sections in mice. Theoretically, it would be possible to accomplish acute brain slice imaging of microglia in rats using fluorescent isolectins (Madry et al. 2018). Is the EGFP signal within microglia in these rats superior to

this approach? One limitation for in vivo imaging of microglia in some mouse models has been the signal intensity from the fluorophore. If possible, the authors should show whether the EGFP signal in these rats is bright enough to resolve the finest small filopodia of microglia (Bernier et al 2019), suggesting

that it will work well for high level in vivo imaging applications (although this would likely require multiphoton in slice imaging approaches).

-We agree with the reviewer that in an ideal scenario, acute slice imaging experiments would be best served by using specific and sophisticated technologies to investigate microglia function in response to physiological perturbation. Our aim, however, was to provide a proof-of-principle in hopes that by demonstrating feasibility, we might embolden other researchers to pursue these interesting questions in ways that we are not equipped to. Moreover, many other excellent research manuscripts have been published demonstrating the microglial physiological response to ATP or laser lesions. Our goal is not to simply replicate those works, but rather to demonstrate that this new transgenic model is capable of capturing similar microglial dynamics.

To a separate point, we are not trying to claim that this transgenic model is superior to other existing models (Iba1-EGFP or CX3CR1-EGFP mouse, for example) or techniques (such isolectin-b4 labeling). We hope that each researcher will consider their experimental questions, available models, and techniques, and make the decision that allows them to best conduct their research. To this end, we simply hope to provide a new transgenic tool to add to the existing toolbox.

In order to allow researchers to better assess whether the Iba1-EGFP rat is suitable for their needs, we have conducted an additional proof-of-principle experiment using in vivo two photon microscopy to observe microglial process dynamics in the currently well-established CX3CR1-EGFP mouse model and in our new Iba1-EGFP rat model (Figure 7G, 7H).

Minor Concerns

1) The numbers in the text for the % of cells obtained by flow cytometry don’t seem to match up with

the numbers in the Figures proper e.g. 9.8% in the text of Fig. 2A but 8.5% in the Figure proper e.t.c. This seems to be the case in several Figure panels. The authors should check to make sure that these

numbers are congruent.

-The numbers presented in the results section are always mean values (e.g. 9.8% in the text for Figure

2A), whereas the figures themselves show representative data from a single experimental sample (e.g.

8.5% for Figure 2A). We have added text to clarify this important point.

2) Several places through the text, the authors although looking only at P60 young adults, use the

phrase “throughout life”. While this is likely true, the authors do not provide evidence for this. Therefore these should be modified to state “into early adulthood” e.g. second paragraph of the Results, the end

of the paragraph on “Histological validation of EGFP+ cells” and the second paragraph of the Discussion. The authors could state “into early adulthood and may do so throughout life”.

-We appreciate the feedback and have changed the text as requested.

3) Although the authors use Iba1 and CX3CR1 (third paragraph of the Results) as “microglia-enriched genes", these genes can be expressed by other border-associated brain macrophages like perivascular macrophages. It would be nice if the authors can also sort or do IHC using P2RY12 which is a more selective microglial marker not expressed by perivascular macrophages (Goldman et al., 2016).

-Our goal with analyzing gene expression after EGFP-FACS was to determine whether other major cell types of the brain (neurons, astrocytes, oligodendrocytes) would be erroneously expressing EGFP and therefore complicate FACS-based experiments. As border associated macrophages also express Iba1, we

show that they (and other hematopoietic cells) also express EGFP. Given that border associated macrophages constitute a very small fraction of the brain’s macrophage population, we are not sure what the proposed experiment adds to the conclusions drawn from the present data.

4) In the fourth paragraph of the discussion, the authors mention the need for GRIIN lenses for in vivo imaging of microglia. This is not NECESSARILY the case. Simple cortical imaging does not require GRIN lenses for two photon imaging. The authors should revise this statement.

-We thank the reviewer for pointing this out and have amended the text as requested and even present

2-photon imaging achieved without the use of a GRIIN lens.

5) In the penultimate sentence of 5th paragraph of Discussion the authors state “...making this screening adaptable to most all flow cytometers”. This would better read “”...making this screening adaptable to most (IF NOT ALL) flow cytometers.

-We have amended the text as requested.

6) The legend of the video fails to detail the application of ATP which it should include.

-We apologize for the oversight and have added in language to indicate the timing and concentration of

ATP application to the legend.

7) In table 2 (adult), it is unexpected that 37% of all brain cells are EGFP/+; this number should be around 10% (i.e., the proportion of microglia in the adult brain)? Also, there seemed to be an increased number of CD11b+/CD45int cells in Iba1-EGFP rats (19% in control vs. 36% in the transgenic rats). Does this indicate ongoing inflammation or resident cell-types in the brain other than microglia or infiltrating cells from the periphery express EGFP? Instead of such a confusing table, it will be intuitive and helpful for readers to see the plots with the individual data points in the figures, and the exact number can be presented in the text of the manuscript.

-As outlined in our methodology, all of our flow cytometry preparations were done with Percoll gradients to enrich for immune cell populations. Thus, the numbers presented are relative, not absolute quantifications, and are presented to demonstrate that the vast majority of immune cells in the brain

are microglia that robustly express EGFP. The data in Table 2 are also present as representative percentages in the flow cytometry gates in Figure 2. We hope that by keeping the summary statistics for the data in table format, readers will be better able to follow the text and focus on the key elements of the (already crowded) figures.

8) The authors should provide FACS scatter plots from the matching control groups (Fig. 3A and D), especially when the authors discuss about a distinct population of CD45/EGFP+ cells in transgenic vs. control rats.

-We have provided representative wild type contour plots for Figure 3, and hope this removes any confusion surrounding the interpretation of the flow cytometry results.

9) Figure 4B indicates that of all EGFP+ cells in the brain, 60% are microglial cells (myeloid/macrophage origin), and the rest 40% are monocytes and other cells. How do these numbers corroborate with the distinct cell-types in the brain expressing Iba1? In general, the information provided in Figure 4 is confusing and requires a table or pie chart to explain which ‘immune’ cell-types are there in the adult rat brain and what proportion of these are EGFP+. It is essential to precisely know the identity of all cell- types being sorted when Iba1-EGFP rats are used.

-As mentioned in the text, the aforementioned figure (now Figure 5 in the revised version of the manuscript) is an analysis of the hematopoietic immune cell compartment (i.e. CD45high cells, not

including microglia) only, in hopes to further delineate the other hematopoietic cell types that may be expressing EGFP in the blood and brain. To clarify this further, we have added “CD45high” to the axis titles of Figure 5A, 5B, and 5C, and have added a pie chart to present an alternative visualization of EGFP+ hematopoietic cells (Figure 5C). These data, taken with the data from Figure 2 and Table 2, demonstrate that as expected, the most abundant EGFP+ immune cells in the brain are microglia (95.3% of EGFP+

cells in neonates, 89.8% of EGFP+ cells in adults), with myeloid/macrophages and monocytes being second and third most abundant, respectively.

10) Throughout the study, the authors used either homozygote or heterozygote rats for experiments. For all experiments, please provide the genotype of transgenic and control rats in all the figures.

-We have added genotype labels to each figure and/or figure legend as requested.

11) The authors mentioned 94.9% of EGFP+ cells were CD11b+/CD45int in Iba1-EGFP rats; later, they refer that myeloid cells/macrophages (CD45high/CD11b+/RT1B+) were the most prevalent cell-type in the brain, comprising 61.7% of EGFP+ cells. Such comparisons are quite confusing and the authors need to clarify this in the text.

-As mentioned above (see response to #9), the myeloid/macrophage prevalence is in relation to the analysis of EGFP expression only in CD45high cells. Thus, they are the most abundant non-microglial (CD45int) EGFP-expressing cell in the brain. We have reworded the text of the Results to clarify this issue.
